# Targeting the ARRDC3–DRP1 Axis via hUMSC‐Derived Exosomal CRYAB for Neuroprotection in Cerebral Ischemia/Reperfusion Injury

**DOI:** 10.1002/adhm.202503803

**Published:** 2026-01-19

**Authors:** Rong ji, Zengyu Zhang, Zhuohang Liu, Kaicheng Yang, Xueyu Mao, Min Chu, Yong Wang, Jing Zhao

**Affiliations:** ^1^ Department of Neurology Minhang Hospital Fudan University Shanghai China; ^2^ Department of Rehabilitation Medicine Zhongshan Hospital Fudan University Shanghai China; ^3^ Department of Geriatrics Shanghai Geriatric Medical Center Shanghai China; ^4^ Department of Neurology Zhongshan Hospital Fudan University Shanghai China; ^5^ Institute of Healthy Yangtze River Delta Shanghai Jiao Tong University Shanghai China

**Keywords:** ARRDC3/DRP1 axis, cerebral ischemia‐reperfusion injury, exosomes, ferroptosis, mitochondrial dynamics

## Abstract

Cerebral ischemia/reperfusion injury (CIRI) remains a major clinical challenge due to the lack of effective neuroprotective strategies. Here, hUMSC‐derived exosomes (H‐Exo) were isolated and administered intranasally (15 µg/mouse/day for 3 days) in a mouse middle cerebral artery occlusion/reperfusion (MCAO/R) model. Animals were randomly assigned to three groups: Sham, MCAO/R, and H‐Exo–treated MCAO/R mice. H‐Exo efficiently penetrated the blood–brain barrier, accumulated within the ischemic penumbra, and was internalized by neurons and glial cells. Treatment with H‐Exo markedly improved neurological function both in vivo and in vitro. Mechanistically, H‐Exo inhibits neuronal ferroptosis by preserving mitochondrial dynamics and alleviating oxidative stress. Transcriptomic analysis identified ARRDC3 as a previously unrecognized ferroptosis‐associated gene that was upregulated after ischemia but suppressed by H‐Exo treatment. ARRDC3 exacerbates neuronal ferroptosis by promoting Drp1‐dependent mitochondrial fragmentation. Proteomic profiling further identified CRYAB as an abundant exosomal cargo mediating the neuroprotective effects of H‐Exo. Pharmacological inhibition of CRYAB with NCI‐41356 partially reversed the anti‐ferroptotic effects of H‐Exo, confirming its essential role. Collectively, this study reveals the CRYAB–ARRDC3–Drp1 axis as a key regulator linking mitochondrial dynamics to ferroptosis and highlights H‐Exo as a promising non‐invasive therapeutic approach for ischemic stroke.

## Graphical abstract

Intranasally administered hUMSC‐derived exosomes modulate the CRYAB–ARRDC3–Drp1 axis, alleviating mitochondrial dysfunction and ferroptosis, enhancing neuronal survival, reducing oxidative stress, and promoting functional recovery in ischemia‐reperfusion injury, offering a promising therapeutic strategy for ischemic stroke.

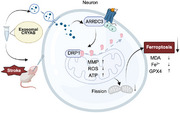

## 1 Introduction

Ischemic stroke continues to pose a major global health burden, affecting approximately 69.9 million individuals and accounting for 3.6 million deaths annually, making it one of the leading causes of mortality and long‐term disability worldwide [1]. It results from an abrupt disruption of cerebral circulation that leads to neuronal injury and subsequent loss of neurological function [2, 3]. Despite advances in recombinant tissue plasminogen activator (rt‐PA) thrombolysis [4] and mechanical thrombectomy [5]. Strict eligibility criteria and reperfusion‐related injury continue to hinder effective treatment of acute ischemic stroke [6]. Thus, novel strategies to enhance post‐stroke recovery are urgently needed.

In recent years, exosome‐based therapy has emerged as an effective strategy for the treatment of ischemic stroke. Mesenchymal stem cell‐derived exosomes (MSC‐Exos) serve as key mediators of intercellular communication by delivering bioactive molecules, including proteins, mRNA, and microRNA, to target cells, thereby precisely modulating their biological functions [7, 8]. As an emerging cell‐free therapy, exosome‐based treatment offers several advantages over traditional stem cell transplantation [9], including a noninvasive production process, low immunogenicity, nanoscale size that facilitates blood–brain barrier penetration, and avoidance of cell transplantation‐related risks such as pulmonary embolism [10, 11]. Owing to these unique properties, exosomes have garnered significant attention as an alternative or adjunct to stem cell therapy [12].

Cerebral ischemia‐reperfusion injury (CIRI) is a complex pathophysiological process involving oxidative stress, energy metabolism disorders, intracellular calcium homeostasis imbalance, inflammatory responses, and multiple forms of cell death, including apoptosis and ferroptosis [13, 14]. Mitochondrial dysfunction plays a crucial role in CIRI and constitutes a major contributor to neuronal loss following ischemic insult [15, 16]. Mitochondria maintain their functionality through dynamic morphological changes, including continuous fusion and division events [17]. In mammalian cells, the key regulator of mitochondrial fission is the GTPase dynamin‐related protein 1 (Drp1), which relocates from the cytoplasm to the outer mitochondrial membrane (OMM). Upon recruitment, Drp1 assembles into ring‐like or helical structures that facilitate constriction of the membrane, ultimately leading to organelle fragmentation [17, 18]. Under conditions of acute ischemic‐hypoxic injury, Drp1 activity is markedly increased, leading to excessive mitochondrial fragmentation and organelle dysfunction [19]. Studies have shown that CIRI activates Drp1 via phosphorylation, promoting excessive fission, cristae disruption, and mitochondrial fragmentation. This results in massive ROS accumulation, mitochondrial membrane potential collapse, and impaired energy metabolism, ultimately triggering a pathological oxidative stress–inflammation cascade that exacerbates neuronal injury [20]. Genetic ablation of Drp1 has been shown to protect neurons from ischemia/reperfusion injury by preventing excessive mitochondrial fission and swelling, independent of the mitophagy pathway [21]. Overall, the regulation of mitochondrial fission by Drp1 represents a crucial determinant of neuronal fate after ischemic insult, underscoring the importance of maintaining mitochondrial homeostasis.

Ferroptosis is an iron‐dependent, regulated form of cell death, characterized biochemically by lipid peroxidation and mitochondrial dysfunction. Its typical morphological features include plasma membrane shrinkage, increased membrane density, and reduced or absent mitochondrial cristae [22]. Ferroptosis has emerged as a promising therapeutic target for ischemic stroke [23]. During ferroptosis, depletion of glutathione (GSH) and inactivation of glutathione peroxidase 4 (GPX4) impair the cell's ability to eliminate phospholipid hydroperoxides, resulting in their pathological accumulation within membrane structures. Concurrently, pro‐oxidant factors are markedly elevated, including the accumulation of ferrous iron (Fe^2^^+^), excessive production of reactive oxygen species (ROS), and generation of lipid peroxidation byproducts such as malondialdehyde (MDA) [24, 25]. As the primary site of intracellular ROS production and a central hub for iron metabolism, mitochondria play an essential role in both the initiation and execution of ferroptosis. Their functional state is closely linked to cellular susceptibility to ferroptosis [26]. Increasing evidence indicates that mitochondrial dysfunction not only disrupts bioenergetic homeostasis but also amplifies oxidative stress and lipid peroxidation, thereby promoting ferroptotic injury. DRP1, a key regulator of mitochondrial fission, has been implicated as a critical mediator of mitochondria‐dependent cell death pathways, including ferroptosis [27]. Recent evidence indicates that Drp1–mediated mitochondrial dysfunction serves as a central event in ischemia‐ and hypoxia‐induced neuronal injury. Aberrant activation of Drp1 disrupts mitochondrial quality control and amplifies oxidative stress, thereby linking impaired mitochondrial dynamics to ferroptotic susceptibility [28]. Together, these findings highlight the pivotal role of Drp1–mediated mitochondrial dysfunction in driving ferroptotic neuronal injury, emphasizing the importance of targeting mitochondrial dynamics as a promising therapeutic strategy for ischemic stroke.

Accumulating evidence suggests that the therapeutic effects of human umbilical cord MSCs (hUMSCs) largely depend on the paracrine release of extracellular vesicles (EVs), with exosomes serving as the primary functional component involved in diverse physiological and pathological processes [29]. Additionally, hUMSC transplantation has been shown to improve neurological function and reduce brain damage in rat models of chronic ischemic stroke, largely via paracrine mechanisms [30]. H‐Exo have demonstrated remarkable efficacy in alleviating inflammation, autoimmune dysregulation, and oxidative stress‐related injury [31, 32, 33]. Notably, recent research has reported that magnetically targeted hUMSC‐Exos (Spion‐Ex) delivered miR‐1228‐5p and inhibited the TRAF6/NOX1 pathway, thereby improving mitochondrial function and cognitive deficits in post‐stroke mice [34], highlighting their promising brain‐targeting therapeutic potential. Despite these encouraging findings, the precise molecular mechanisms by which H‐Exo regulate mitochondrial homeostasis and ferroptosis to exert neuroprotective effects in ischemic brain injury remain elusive and warrant further investigation.

The clinical translation of exosome‐based therapy is progressing steadily, with several early‐phase clinical trials initiated worldwide to evaluate the safety and feasibility of MSC‐Exos in neurological disorders. Among them, NCT05158101 is an ongoing Phase I trial investigating the safety of intranasal administration of exosomes derived from allogeneic adult umbilical cord mesenchymal stem cells in patients with ischemic stroke; NCT06995625 is another ongoing Phase I study aiming to assess the safety and tolerability of intravenously administered MSC‐derived exosome therapy in patients with acute ischemic stroke. In addition, a Phase I/II clinical trial (NCT03384433) is currently recruiting patients with acute ischemic stroke to evaluate the safety and efficacy of intravenously administered miR‐124‐modified bone marrow MSC‐derived exosomes (BM‐MSC‐Exos), while NCT07143786 aims to explore the therapeutic potential of exosomes derived from human induced neural stem cells in acute ischemic stroke. Together, these ongoing and planned trials highlight the accelerating global efforts to translate exosome‐based therapies into clinical practice, particularly in the treatment of ischemic stroke, where exosomes may offer a safe, innovative, and accessible therapeutic strategy beyond current treatment limitations.

**SCHEME 1:**
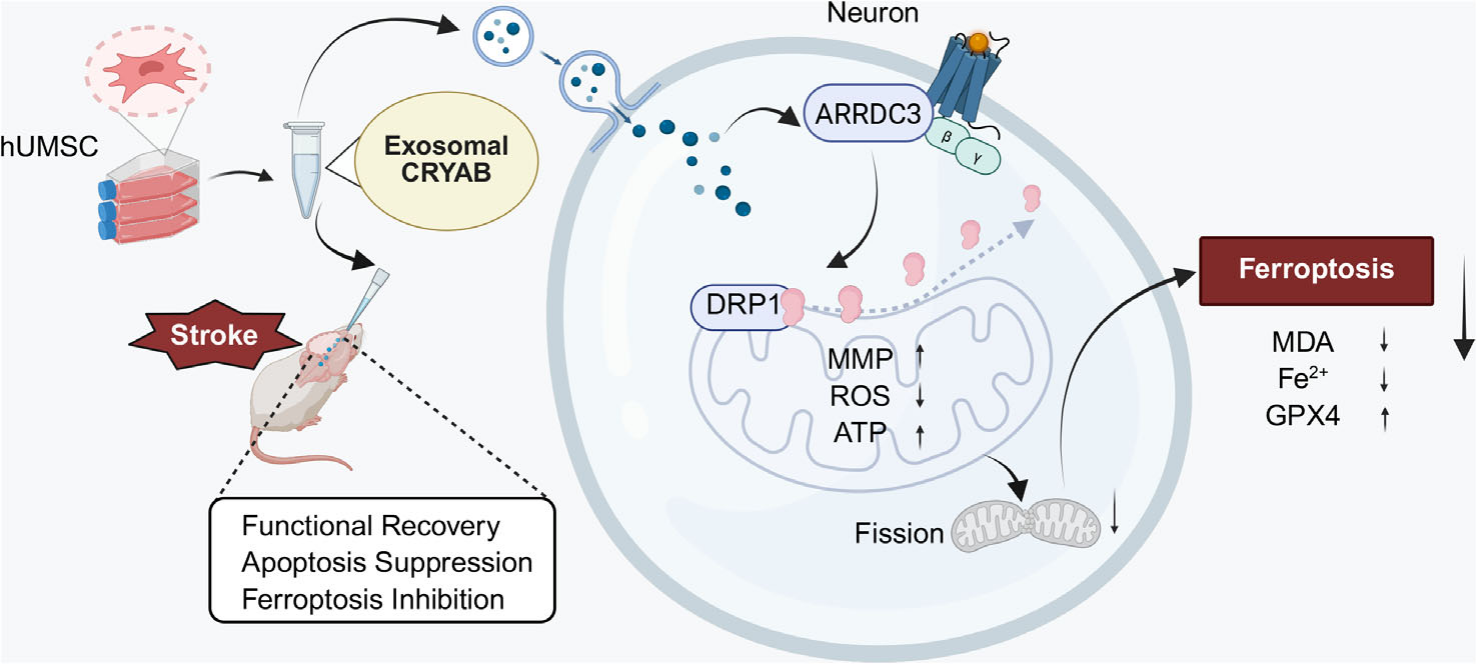
Schematic illustration of the H‐Exo–mediated functional signaling pathway in neurons. This schematic was created with BioRender.com.

The core objective of this study was to systematically evaluate the neuroprotective potential of H‐Exo and to elucidate their underlying molecular mechanisms in both the t‐MCAO and cellular OGD/R models of ischemia. We hypothesized that the protective effects of H‐Exo are critically mediated by maintaining mitochondrial homeostasis, thereby inhibiting neuronal ferroptosis. Building on this, we further investigated whether H‐Exo suppressed ischemia‐induced excessive mitochondrial fission through regulation of a novel signaling pathway—the ARRDC3/Drp1 axis—thus clarifying a key anti‐ferroptotic mechanism. Moreover, proteomic analysis identified the chaperone protein CRYAB as a key bioactive cargo enriched in H‐Exo, which may play a central role in mediating these neuroprotective effects (Scheme 1). Collectively, this study provides new mechanistic insights into how H‐Exo preserve mitochondrial integrity and inhibit neuronal ferroptosis following ischemic insult.

## 2 Results

### 2.1 H‐Exo Isolation, Characterization, and In Vivo Tracing

**FIGURE 1:**
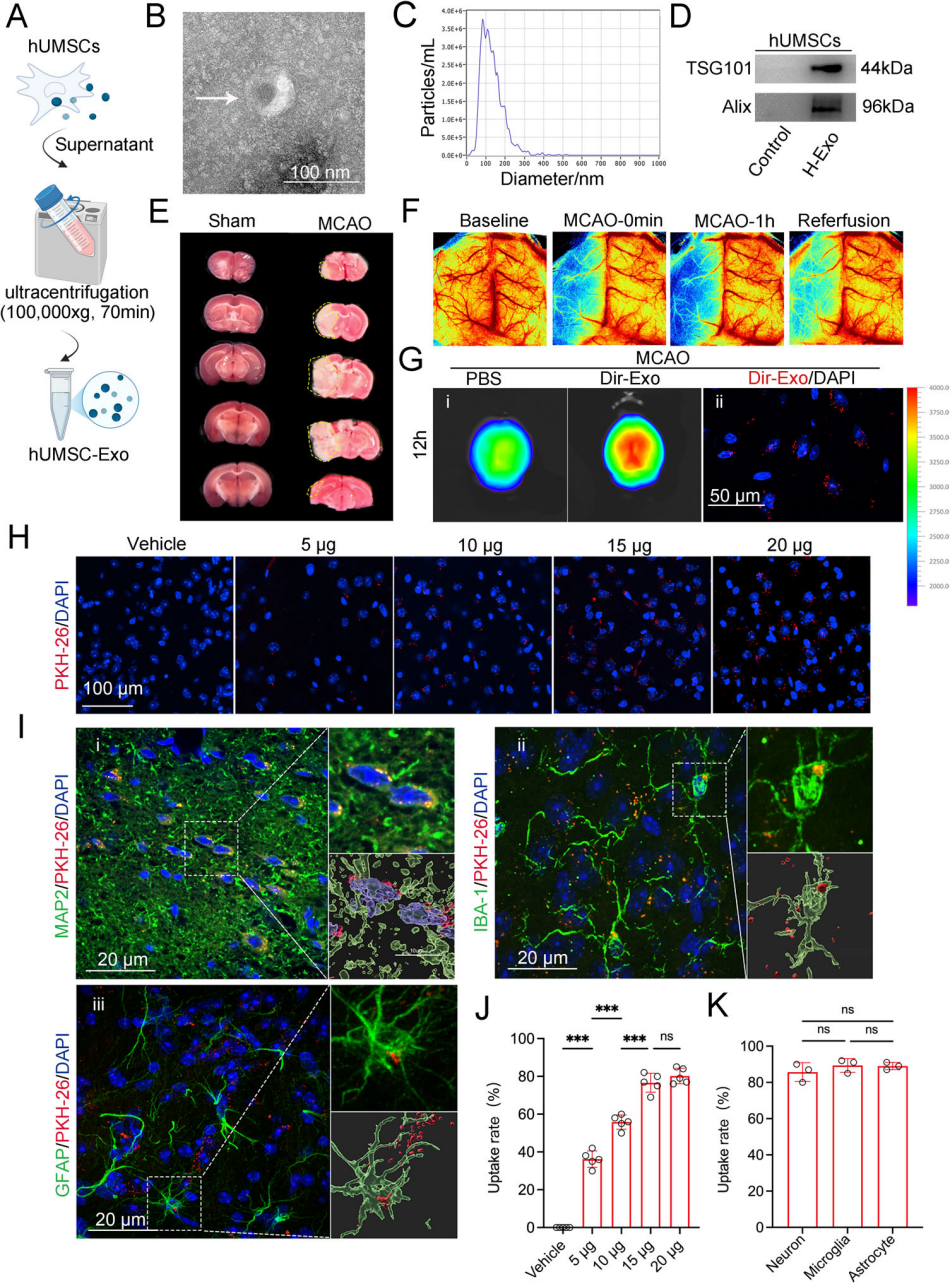
H‐Exo were successfully characterized and tracked in vivo and exhibited robust uptake by neurons, microglia, and astrocytes within the ischemic penumbra. (A) Schematic illustrating the isolation of H‐Exo by ultracentrifugation. This figure was created with BioRender.com. (B) Transmission electron microscopy (TEM) image showing the typical cup‐shaped morphology of H‐Exo (white arrow). Scale bar = 100 nm. (C) Nanoparticle tracking analysis (NTA) demonstrating a predominant particle size distribution around 100 nm. (D) Western blot analysis confirming the expression of exosomal markers Alix and TSG101. (E) Representative TTC staining of brain infarcts. Yellow boxes indicate infarcted regions. (F) Laser speckle contrast imaging (LSCI) showing changes in cerebral blood flow at baseline, during MCAO, and after reperfusion. (G) Representative in vivo fluorescence imaging (i) and immunofluorescence microscopy (ii) performed 12 h after intranasal administration of DiR‐labeled H‐Exo following MCAO. Blue: DAPI. The pseudocolor bar indicates fluorescence intensity in arbitrary units (a.u.). (H,J) Representative images (H) and corresponding quantification (J) of PKH26‐labeled H‐Exo uptake in the ischemic penumbra at different doses on day 3 post‐MCAO. Scale bars = 100 µm. *n* = 5 mice per group. (I,K) Representative immunofluorescence images and Imaris reconstructions showing uptake of exosomes by neurons (i, MAP2, green), microglia (ii, IBA1, green), and astrocytes (iii, GFAP, green) (I), along with quantification of uptake efficiency across cell types (K). Scale bars = 20 µm. *n* = 3 mice per group. One‐way ANOVA; data are presented as mean ± SD. ^***^ *p* < 0.001.

H‐Exo were purified by ultracentrifugation (Figure 1A). Transmission electron microscopy (TEM) revealed that the particles exhibited a typical exosomal morphology, appearing as round or cup‐shaped vesicles (Figure 1B). Nanoparticle tracking analysis (NTA) showed that the particle size was predominantly distributed around 100 nm (Figure 1C). Western blot analysis further confirmed the successful exosome isolation, as the expression levels of the exosomal marker proteins TSG101 (44 kDa) and Alix (96 kDa) were markedly higher in H‐Exo than in the corresponding cell lysate control (Figure 1D).

To evaluate the therapeutic potential of H‐Exo in ischemic brain injury, a transient middle cerebral artery occlusion (tMCAO) model was established in mice. The success of the cerebral ischemia–reperfusion model was verified by TTC staining, which revealed well‐defined infarcted regions (Figure 1E), and by laser speckle contrast imaging, which demonstrated a marked reduction in cerebral perfusion following occlusion and its recovery after reperfusion (Figure 1F).

To assess whether H‐Exo could cross the blood–brain barrier (BBB) and reach the ischemic penumbra, 10 µg of DiR‐labeled H‐Exo (DiR‐Exo) was intranasally administered immediately after reperfusion. In vivo fluorescence imaging at 12 h post‐administration revealed markedly stronger signals in the brains of DiR‐Exo–treated mice than in PBS‐treated controls (Figure 1G_i), suggesting efficient BBB penetration. Immunofluorescence analysis of brain sections further confirmed the internalization of H‐Exo by cells within the ischemic penumbra (Figure 1G_ii). To determine the optimal intranasal dose, mice received escalating doses of PKH26‐labeled H‐Exo (5, 10, 15, and 20 µg) post‐tMCAO. Fluorescence imaging of the ischemic penumbra showed a dose‐dependent increase in exosome uptake by brain cells, with maximal uptake observed at 15 µg (*p* < 0.001; Figure 1H,J), which was therefore selected for subsequent in vivo experiments. Further colocalization analysis confirmed that H‐Exo at this dose were efficiently internalized by neurons (MAP2^+^), microglia (IBA1^+)^, and astrocytes (GFAP^+^) in the ischemic penumbra (Figure 1I). Quantitative analysis revealed high uptake rates of approximately 85%–90% across all three cell types, with no significant differences among them (Figure 1K). Collectively, these findings establish a reliable protocol for intranasal delivery of H‐Exo and demonstrate their efficient BBB penetration and broad cellular uptake within the ischemic penumbra, providing a robust foundation for evaluating their neuroprotective efficacy in subsequent studies.

### 2.2 H‐Exo Alleviated Neuronal Damage and Improved Neurological Function In Vivo

**FIGURE 2:**
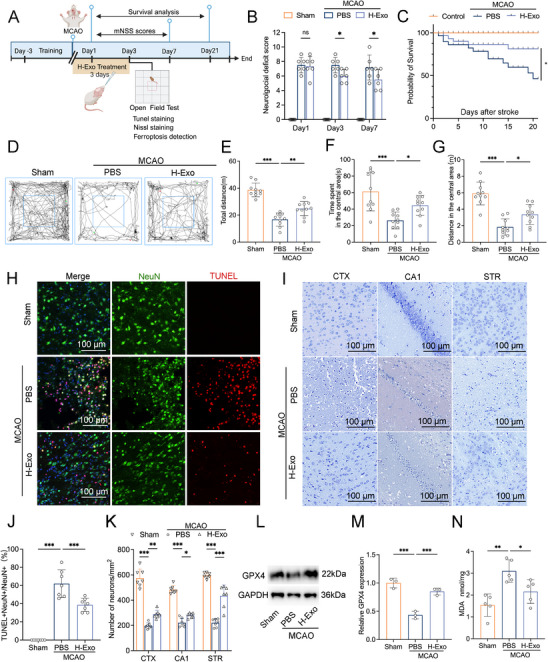
H‐Exo alleviates neurological deficits and inhibits ferroptosis in vivo. (A) Schematic diagram of the experimental design. Mice underwent tMCAO and received intranasal administration of H‐Exo (15 µg) once daily for three consecutive days. Behavioral testing, tissue collection, and ferroptosis evaluation were performed at the indicated time points. This figure was created with BioRender.com. (B) Neurological function was evaluated by mNSS on days 1, 3, and 7 post‐ischemia. *n* = 6 mice per group. (C) Kaplan–Meier survival analysis over a 21 day period following stroke. *n* = 15 mice per group. (D–G) Open field test assessing locomotor and anxiety‐like behaviors, including representative movement traces (D), total distance traveled (E), time spent in the central area (F), and distance traveled in the central area (G). *n* = 10 mice per group. (H J) Representative TUNEL/NeuN immunofluorescence staining (H) and corresponding quantification of TUNEL^+^NeuN^+^/total NeuN^+^ neurons (J), showing reduced neuronal apoptosis in the H‐Exo group. NeuN (green), TUNEL (red). Scale bars = 100 µm. *n* = 7 mice per group. (I,K) Representative Nissl staining of cortex (CTX), hippocampal CA1, and striatum (STR) (I), and quantification of surviving neurons per mm^2^ (K), revealing H‐Exo–mediated neuroprotection across multiple brain regions. Scale bars = 100 µm. *n* = 7 mice per group. (L,M) Western blot analysis (L) and densitometric quantification (M) of GPX4 protein expression. *n* = 3 mice per group. N) Quantification of malondialdehyde (MDA) levels, indicating attenuated lipid peroxidation following H‐Exo treatment. *n* = 5 mice per group. Statistical comparisons in (B,K) were performed using two‐way ANOVA; all others used one‐way ANOVA; Data are presented as mean ± SD. ^*^ *p* < 0.05; ^**^ *p* < 0.01; ^***^ *p* < 0.001.

As shown in Figure 2A, a transient middle cerebral artery occlusion (tMCAO) model was established in mice, followed by intranasal administration of 15 µg H‐Exo immediately after reperfusion and once daily for three consecutive days. Neurological function was evaluated on days 1, 3, and 7 after ischemia, and survival was monitored until day 21. Modified neurological severity scores (mNSS) indicated that H‐Exo treatment significantly improved neurological function on days 3 and 7 compared with the PBS group (*p* < 0.05 for both; Figure 2B). H‐Exo treatment significantly increased survival after ischemia/reperfusion compared with the PBS group, suggesting a protective role of H‐Exo in reducing post‐stroke mortality (*p* < 0.05; Figure 2C).

To further assess motor activity and anxiety‐like behavior, an open field test (OFT) was performed. As shown in Figure 2D, H‐Exo treatment significantly increased the total distance traveled (*p* < 0.01), as well as the time spent (*p* < 0.001) and distance traveled in the central area (*p* < 0.05) compared with PBS (Figure 2E–G), thereby indicating improved motor performance and reduced anxiety‐like behavior. To determine whether this behavioral improvement correlated with reduced neuronal injury, TUNEL/NeuN immunofluorescence staining was performed at 72 h post‐reperfusion, which revealed a significant reduction in the number of TUNEL‐positive neurons in the H‐Exo group compared with the PBS group (*p* < 0.001; Figure 2H,J), suggesting effective suppression of ischemia‐induced neuronal death. Consistently, Nissl staining demonstrated significant neuronal loss and morphological damage in the cortex (CTX), hippocampal CA1 region, and striatum (STR) following MCAO, whereas H‐Exo treatment substantially preserved neuronal structure and mitigated cell loss in all three regions (CTX: *p* < 0.01; CA1: *p* < 0.05; STR: *p* < 0.001; Figure 2I,K). To further delineate the neuroprotective spectrum of H‐Exo, we examined the CA3 and entorhinal cortex (Ect) regions and observed that H‐Exo markedly alleviated neuronal loss in both areas (Figure S1A,B), further supporting its broad neuroprotective effect. Given our previous observation that ferroptosis peaks at day 3 post‐MCAO [23], this time point was selected for subsequent mechanistic analysis. Western blot analysis showed that H‐Exo markedly upregulated glutathione peroxidase 4 (GPX4), a critical regulator of ferroptosis (*p* < 0.001; Figure 2L,M), and downregulated malondialdehyde (MDA) levels, a marker of lipid peroxidation (*p* < 0.05 vs PBS; Figure 2N), indicating that H‐Exo effectively attenuated cerebral ferroptosis. In summary, these results demonstrate that H‐Exo exerts potent neuroprotective effects against ischemic brain injury, likely through promoting neurological recovery, reducing anxiety‐like behaviors, and inhibiting neuronal ferroptosis.

### 2.3 H‐Exo Suppress Neuronal Ferroptosis by Regulating Drp1–Mediated Mitochondrial Dynamics Abnormalities

**FIGURE 3:**
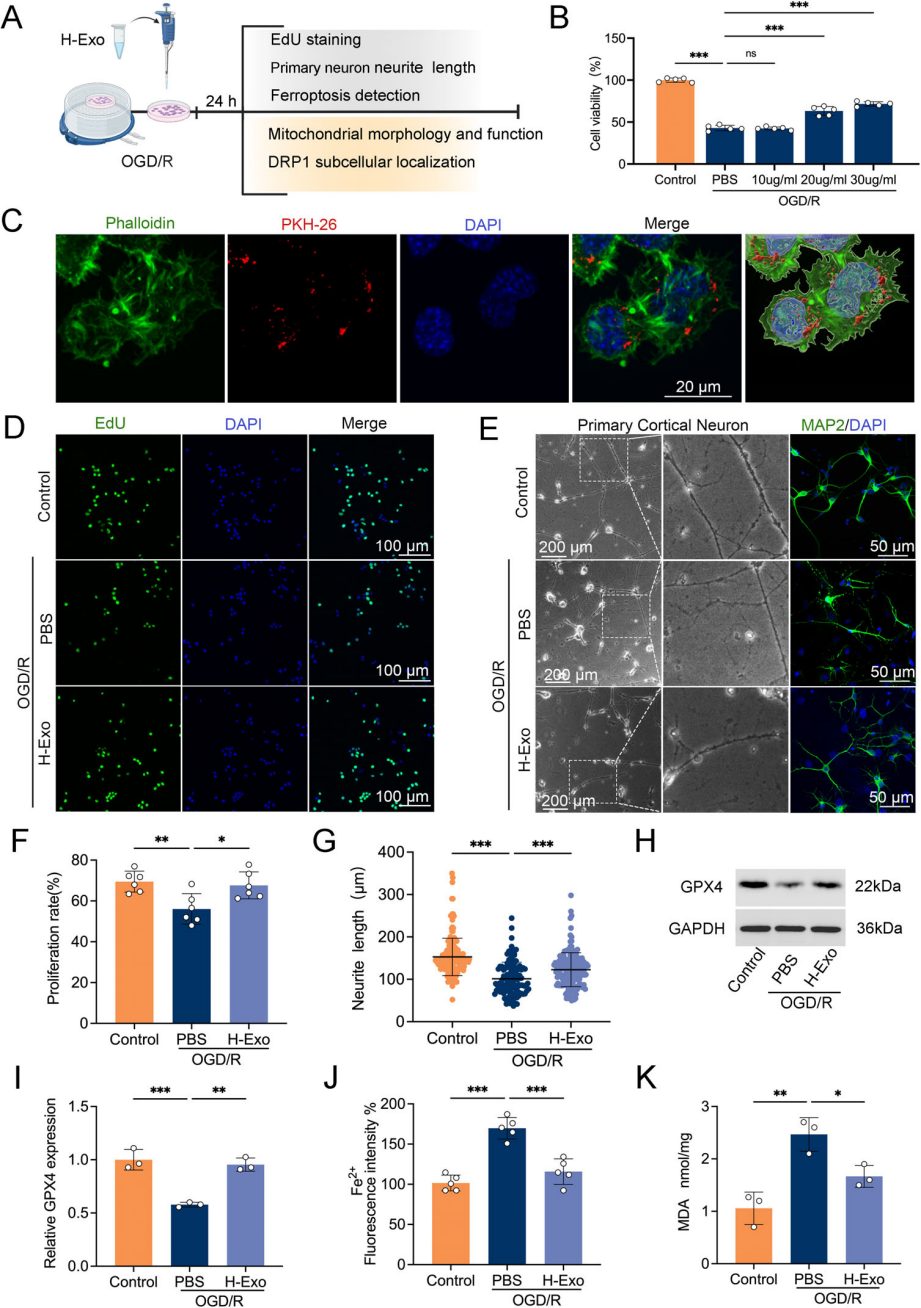
H‐Exo promotes neuroprotection and suppresses neuronal ferroptosis in vitro. (A) Schematic illustration of the in vitro experimental design. N2a cells and primary cortical neurons were subjected to OGD/R and treated with H‐Exo for 24 h after reoxygenation. This figure was created with BioRender.com. (B) Cell viability of OGD/R‐injured N2a cells treated with various concentrations of H‐Exo, showing a dose‐dependent improvement. *n* = 5 independent experiments. (C) Representative fluorescence images and 3D reconstructions using Imaris software showing the internalization of PKH26‐labeled H‐Exo (red) by N2a cells stained with phalloidin (green) and DAPI (blue). Scale bar = 20 µm. (D,F) Representative EdU/DAPI staining images (D) and quantification (F) of proliferating N2a cells. Scale bars = 100 µm. *n* = 6 independent experiments. (E,G) Representative bright‐field and MAP2 immunofluorescence images (E) of primary cortical neurons showing neurite morphology, with corresponding quantification of neurite length (G). Scale bars = 200 µm (bright‐field) and 50 µm (MAP2/DAPI). *n* = 50–60 neurons per group from three independent cultures. (H,I) Western blot analysis (H) and quantification (I) of GPX4 expression in N2a cells. *n* = 3 independent experiments. (J) Quantification of intracellular Fe^2^^+^ fluorescence intensity using FerroOrange. *n* = 5 independent experiments. (K) Quantification of MDA levels. *n* = 3 independent experiments. Statistical analysis was performed using one‐way ANOVA. Data are presented as mean ± SD. ^*^ *p* < 0.05; ^**^ *p* < 0.01; ^***^ *p* < 0.001.

To further evaluate the neuroprotective effects of H‐Exo in vitro, an oxygen–glucose deprivation/reoxygenation (OGD/R) model was established using N2a cells and primary cortical neurons (Figure 3A). After 4 h of OGD, N2a cells were treated with various concentrations of H‐Exo, and cell viability was assessed 24 h later. Cell viability improved in a dose‐dependent manner, with 20 µg/mL identified as the optimal concentration (Figure 3B), which was therefore used for subsequent experiments. To confirm cellular uptake of H‐Exo, PKH26‐labeled H‐Exo was co‐cultured with N2a cells for 6 h after OGD/R. Fluorescence microscopy revealed efficient internalization of H‐Exo (Figure 3C), which was further visualized by 3D reconstruction using Imaris software. In addition, H‐Exo significantly promoted the proliferation of OGD/R‐injured N2a cells compared with the PBS group (*p* < 0.05; Figure 3D,F). In primary cortical neurons, H‐Exo treatment markedly rescued neurite outgrowth impaired by OGD/R, as evidenced by MAP2 immunostaining (*p* < 0.001; Figure 3E,G).

Given these protective effects, we further examined markers of ferroptosis. Western blot analysis showed that H‐Exo significantly upregulated GPX4 expression in OGD/R‐injured cells compared with the PBS group (*p* < 0.01; Figure 3H,I). Correspondingly, H‐Exo treatment markedly reduced intracellular Fe^2^^+^ accumulation (*p* < 0.001; Figure 3J) and suppressed MDA production (*p* < 0.01; Figure 3K), indicating that H‐Exo effectively attenuates OGD/R‐induced ferroptosis.

**FIGURE 4:**
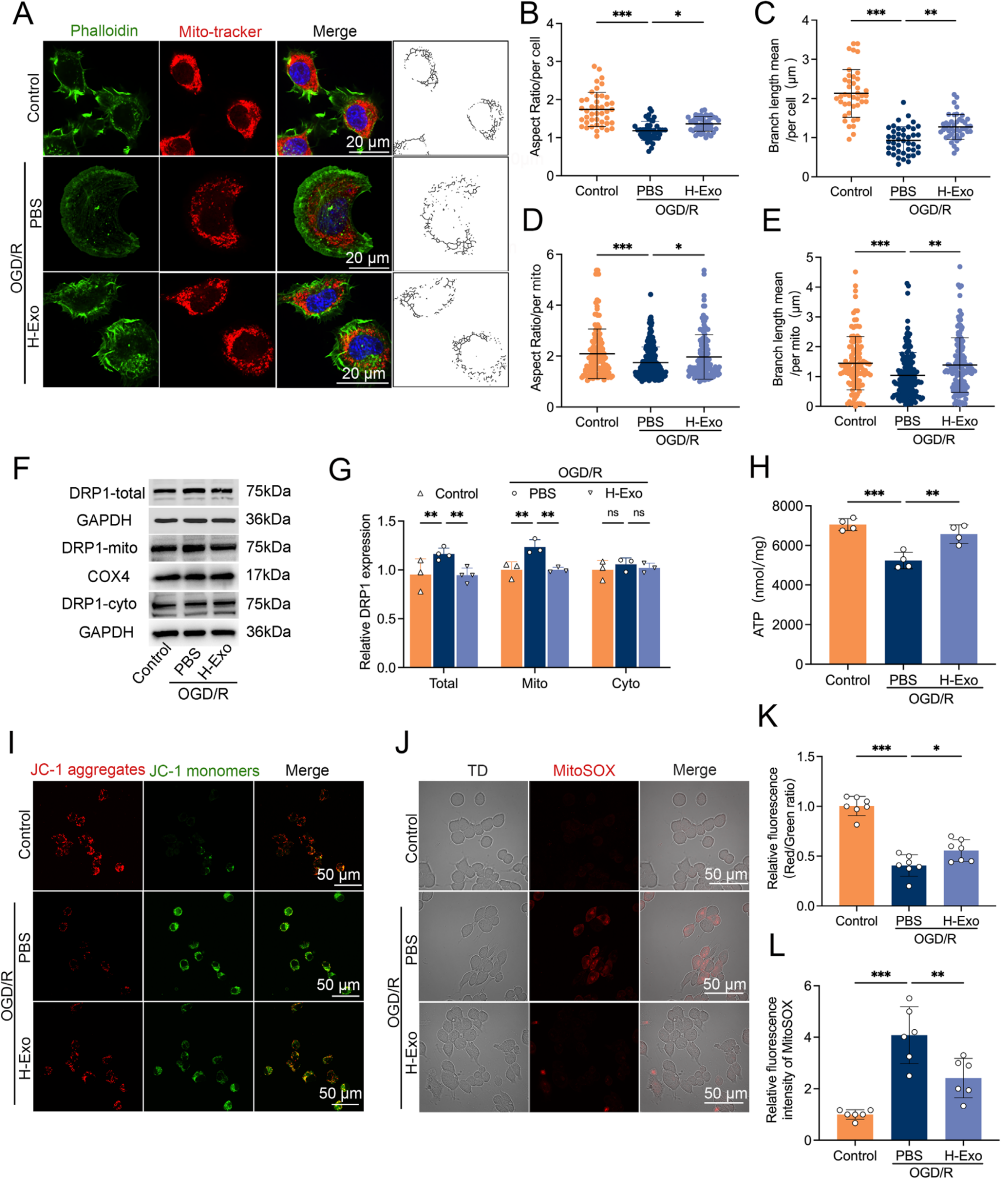
H‐Exo alleviates OGD/R–induced mitochondrial fragmentation and dysfunction by modulating DRP1 activation in vitro. (A) Representative fluorescence images of N2a cells stained with phalloidin (green) and MitoTracker Red CMXRos (red). Merged images and the corresponding binary skeletonization (rightmost) show mitochondrial morphology. Scale bars = 20 µm. (B–E) Quantitative analysis of mitochondrial morphology, including aspect ratio and mean branch length of per cell (B,C) and per mitochondrion (D,E), measured using the Mitochondria Analyzer plugin in Fiji. *n* = 40–50 cells and 150–300 mitochondria per group, collected from three independent experiments. (F,G) Western blot analysis (F) and quantification (G) of DRP1 expression in total, mitochondrial, and cytosolic fractions. COX4 was used as a mitochondrial loading control, and GAPDH as a cytosolic control. *n* = 3 independent experiments. (H) Quantification of ATP production. (I,K) Representative JC‐1 staining images (I) and quantification (K) showing mitochondrial membrane potential (MMP). Scale bars = 50 µm. (J,L) Representative MitoSOX (red) staining images (J) and quantification (L) showing mitochondrial reactive oxygen species (ROS) levels. Scale bar = 50 µm. Statistical comparisons in (G) were performed using two‐way ANOVA; all others used one‐way ANOVA. Data are presented as mean ± SD. ^*^ *p* < 0.05; ^**^ *p* < 0.01; ^***^ *p* < 0.001.

Mitochondrial dysfunction represents a central pathological mechanism underlying both CIRI and ferroptosis, and inhibition of excessive mitochondrial fission has been reported to inhibit ferroptosis and mitigate CIRI severity [35]. To assess the effects of H‐Exo on mitochondrial dynamics, mitochondria were stained with MitoTracker Red CMXRos and skeletonized using Fiji (Figure 4A). Morphological parameters were quantitatively analyzed using the Mitochondria Analyzer plugin. OGD/R exposure induced pronounced mitochondrial fragmentation, as evidenced by significant reductions in both the aspect ratio and mean branch length at the cellular level (both *p* < 0.001; Figure 4B,C), as well as in individual mitochondria (both *p* < 0.001; Figure 4D,E). Notably, H‐Exo treatment markedly restored mitochondrial morphology, reversing these OGD/R‐induced abnormalities (Figure 4A–E). The aspect ratio reflects mitochondrial elongation, whereas mean branch length indicates the extent of mitochondrial network connectivity. These findings suggest that H‐Exo preserves mitochondrial structural integrity and mitigates OGD/R‐induced mitochondrial fragmentation.

Given the central role of DRP1 in regulating mitochondrial fission, we next investigated whether the protective effects of H‐Exo involve the modulation of DRP1 activation and subcellular localization. Western blot analysis demonstrated that OGD/R markedly increased both total DRP1 and mitochondrial DRP1 levels (both *p* < 0.01), whereas cytosolic DRP1 expression remained unchanged (Figure 4F,G). Importantly, H‐Exo treatment significantly reduced both total and mitochondrial DRP1 expression compared with the PBS group (both *p* < 0.05; Figure 4F,G). These molecular alterations were accompanied by improved mitochondrial function, as evidenced by increased ATP production (*p* < 0.001 vs PBS; Figure 4H), restoration of mitochondrial membrane potential (MMP; *p* < 0.001 vs PBS; Figure 4I,K), and reduced mitochondrial reactive oxygen species (ROS) levels (*p* < 0.01 vs PBS; Figure 4J,L). Collectively, these findings suggest that H‐Exo preserves mitochondrial homeostasis by regulating DRP1–mediated dynamics, thereby attenuating neuronal ferroptosis.

### 2.4 RNA‐seq Analysis Identifies ARRDC3 as a Key Target of H‐Exo Against Neuronal Ferroptosis

**FIGURE 5:**
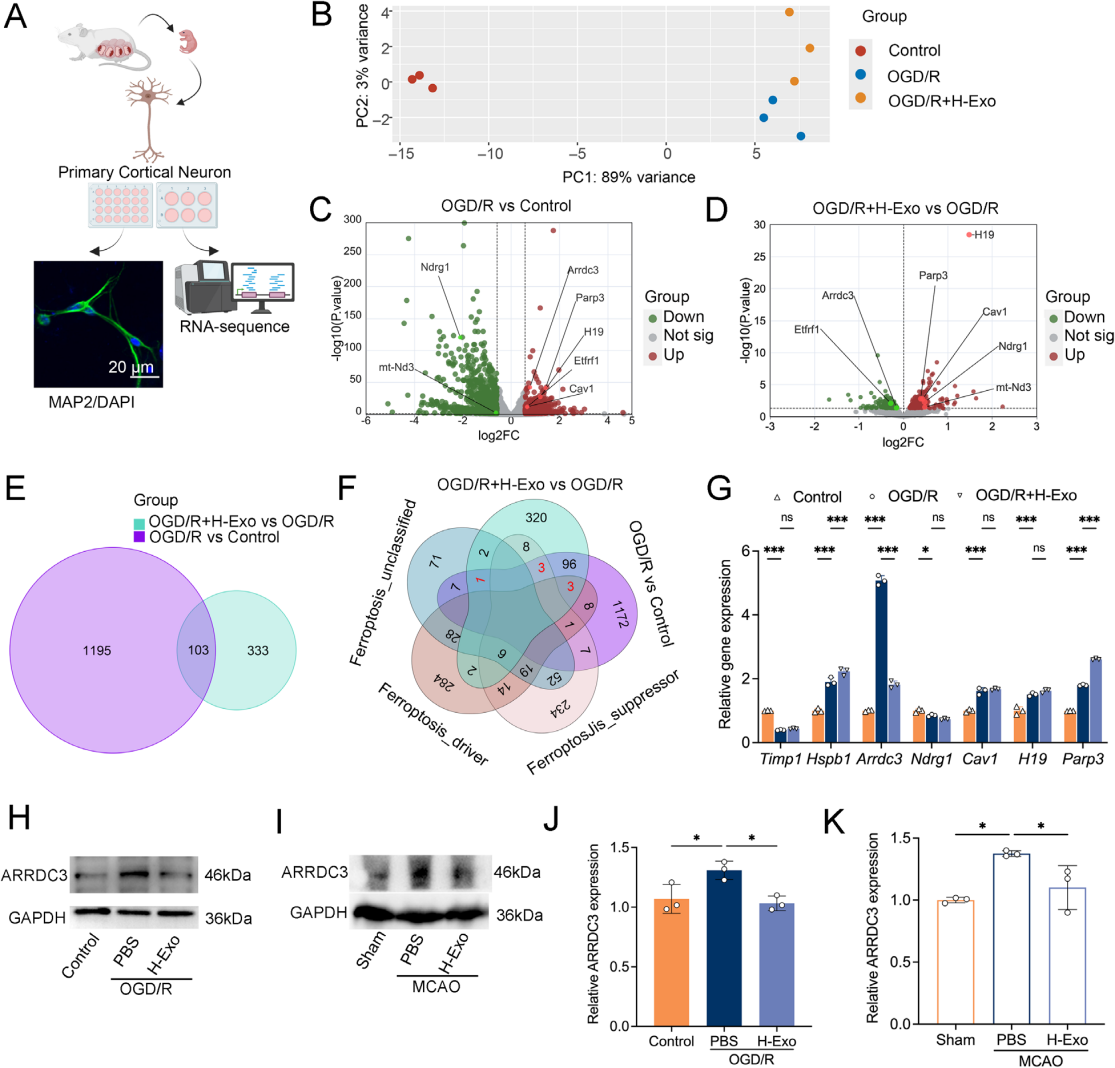
Transcriptomic and molecular analyses identify *ARRDC3* as a key ferroptosis‐related target mediating H‐Exo–induced neuroprotection. (A) Schematic illustration of the workflow for primary cortical neuron isolation, OGD/R model establishment, H‐Exo intervention, and subsequent RNA sequencing. This figure was created with BioRender.com. Representative immunofluorescence image showing MAP2 (green) and DAPI (blue) in cultured neurons. Scale bar = 20 µm. (B) Principal component analysis (PCA) showing distinct transcriptomic separation among Control, OGD/R, and OGD/R + H‐Exo groups. (C,D) Volcano plots displaying differentially expressed genes (DEGs) between OGD/R vs Control (C) and OGD/R + H‐Exo vs OGD/R (D) groups. (E) Venn diagram showing the overlap of DEGs between the OGD/R vs Control and OGD/R + H‐Exo vs OGD/R comparisons. (F) Venn diagram intersecting DEGs with ferroptosis‐related gene sets (drivers, suppressors, and unclassified genes). (G) qRT‐PCR validation of the seven candidate ferroptosis‐associated genes across groups. (H,I) Western blot analysis of ARRDC3 protein expression in vitro (H, OGD/R model) and in vivo (I, MCAO model). (J,K) Quantification of ARRDC3 protein levels in the respective OGD/R (J) and MCAO (K) models*. n* = 3 independent experiments; *n* = 3 mice per group. Statistical comparisons in (G) were performed using two‐way ANOVA; all others used one‐way ANOVA. Data are presented as mean ± SD. ^*^ *p* < 0.05; ^***^ *p* < 0.001.

To elucidate the molecular mechanisms underlying H‐Exo–mediated neuroprotection against ferroptosis, RNA sequencing was performed on primary cortical neurons isolated from fetal mice under three experimental conditions: Control, OGD/R, and OGD/R + H‐Exo (Figure 5A). Principal component analysis (PCA) revealed distinct transcriptomic profiles with clear separation among the three groups (Figure 5B). Differential gene expression (DGE) analysis revealed extensive transcriptomic alterations induced by OGD/R injury. Compared with the Control group, OGD/R resulted in significant changes in 1298 genes (|log_2_FC| > 0.58, P < 0.05), including 426 upregulated and 872 downregulated genes. Volcano plots visualized the global distribution patterns of these DEGs (Figure 5C,D). Notably, H‐Exo treatment significantly modulated 436 genes, including 258 upregulated and 178 downregulated genes (Figure 5C,D), suggesting that H‐Exo exerts its neuroprotective effects through transcriptional reprogramming. To identify ferroptosis‐related genes regulated by H‐Exo, DEGs were intersected with curated ferroptosis gene databases. Venn diagram analysis identified 103 overlapping genes shared between the OGD/R vs Control and OGD/R + H‐Exo vs OGD/R comparisons (Figure 5E). Further intersection with ferroptosis‐related gene databases identified seven key ferroptosis‐associated genes, including three drivers (*H19, TIMP1, NDRG1*), three suppressors (*HSPB1, PARP3, CAV1*), and one unclassified regulator (*ARRDC3*) (Figure 5F).

To validate these transcriptomic findings, qRT‐PCR was performed on the seven ferroptosis‐related candidate genes. Among them, only *ARRDC3* exhibited a consistent bidirectional regulation pattern—being upregulated in OGD/R neurons (*p* < 0.001) and significantly suppressed by H‐Exo treatment (*p* < 0.01; Figure 5G). The other candidate genes did not show consistent or statistically significant expression changes upon validation (Figure 5G). Western blot analysis further confirmed these findings at the protein level, consistent with the mRNA expression patterns observed both in vitro and in vivo (*p* < 0.01 and *p* < 0.05, respectively; Figure 5H–K). Collectively, the integrated transcriptomic and molecular analyses identified *ARRDC3* as the sole ferroptosis‐related gene consistently regulated across ischemia–reperfusion models, indicating its pivotal role in H‐Exo–mediated neuroprotection.

### 2.5 H‐Exo Against Ferroptosis by Regulating ARRDC3/DRP1–Mediated Mitochondrial Fission in OGD/R‐Injured Neurons

**FIGURE 6:**
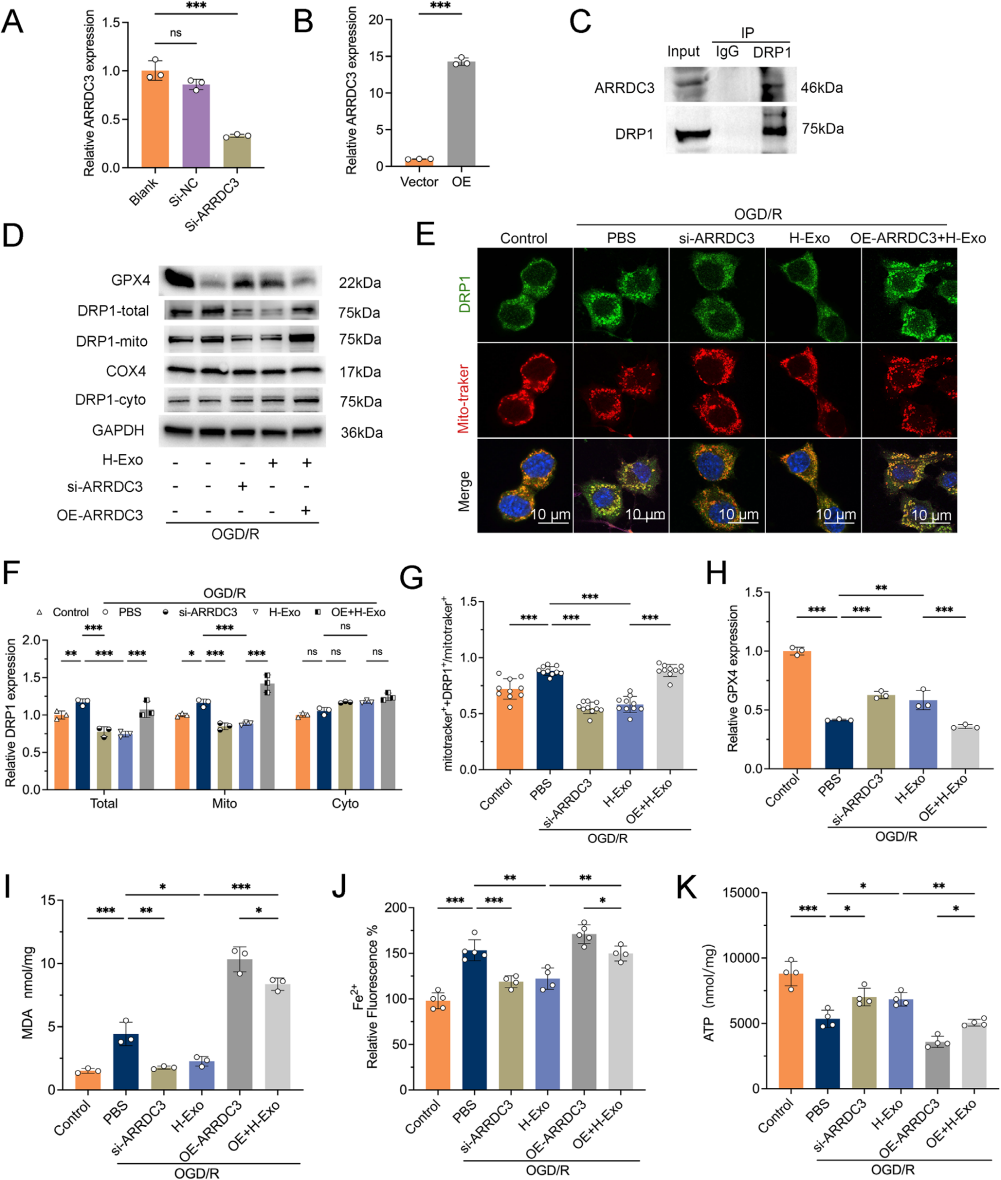
H‐Exo alleviates mitochondrial ferroptosis by modulating the ARRDC3/DRP1 axis in vitro. (A,B) Validation of *ARRDC3* knockdown and overexpression efficiency by qRT‐PCR. *n* = 3 independent experiments. (C) Co‐immunoprecipitation (Co‐IP) analysis of the interaction between ARRDC3 and DRP1. (D,F,H) Western blot analysis of GPX4, total DRP1, mitochondrial DRP1, and cytosolic DRP1 expression in OGD/R‐injured neurons under various treatments, with quantification of DRP1 (F) and GPX4 (H) protein levels. *n* = 3 independent experiments. (E,G) Representative immunofluorescence images showing DRP1 (green), mitochondria stained with MitoTracker (red), and nuclei with DAPI (blue), along with quantification of DRP1 colocalization with mitochondria (G). Scale bars = 10 µm. For (G), each dot represents one image field; *n* = 10 image fields from three independent experiments. (I–K) Quantification of MDA levels (I; *n* = 3 independent experiments), intracellular Fe^2^^+^ accumulation (J; *n* = 4 independent experiments), and ATP production (K; *n* = 4 independent experiments). Statistical comparisons in (B) were performed using *t*‐test, and those in (F) were performed using two‐way ANOVA; all others used one‐way ANOVA. Data are presented as mean ± SD. ^*^ *p* < 0.05; ^**^ *p* < 0.01; ^***^ *p* < 0.001.

ARRDC3 (arrestin domain–containing protein 3), also known as TLIMP‐like inducible membrane protein (TLIMP), is a member of the recently identified mammalian α‐arrestin family. Proteomic studies have revealed that ARRDC3 interacts with a wide spectrum of proteins, including kinases, phosphatases, E3 ubiquitin ligases, deubiquitinases, adaptor proteins, scaffold proteins, and other regulatory molecules, indicating its involvement in diverse cellular processes and signaling pathways [36]. To further investigate the role of ARRDC3 in regulating neuronal mitochondrial fission and ferroptosis, we performed functional studies by knocking down or overexpressing ARRDC3 in N2a cells. Both qRT‐PCR and Western blot confirmed the efficiency of these genetic manipulations (Figure 6A,B; Figure S2A–D). Co‐immunoprecipitation experiments were performed to investigate the interaction between ARRDC3 and DRP1. Western blot analysis revealed that ARRDC3 was detected in the DRP1 immunoprecipitate, suggesting a potential interaction between ARRDC3 and DRP1 (Figure 6C). Analysis of DRP1 levels in total, mitochondrial, and cytosolic fractions showed that ARRDC3 knockdown significantly suppressed the OGD/R‐induced increase in total and mitochondrial DRP1 (both *p* < 0.001 vs. PBS; Figure 6D,F), whereas ARRDC3 overexpression reversed the inhibitory effect of H‐Exo on DRP1 expression (*p* < 0.001; Figure 6D,F). No significant changes were observed in cytosolic DRP1 among the groups (Figure 6D,F). Immunofluorescence staining further confirmed these findings. OGD/R increased DRP1 mitochondrial localization, as indicated by its colocalization with MitoTracker (*p* < 0.001 vs Control; Figure 6E,G). This effect was significantly attenuated by ARRDC3 knockdown or H‐Exo treatment (*p* < 0.001 vs PBS for both; Figure 6E,G), while ARRDC3 overexpression reversed the inhibitory effect of H‐Exo (*p* < 0.001 vs H‐Exo; Figure 6E,G).

Regarding ferroptosis markers, ARRDC3 knockdown markedly restored GPX4 expression, which had been suppressed by OGD/R (*p* < 0.001 vs PBS; Figure 6D,H). H‐Exo treatment also significantly increased GPX4 levels (*p* < 0.01 vs PBS), whereas ARRDC3 overexpression abolished this effect (*p* < 0.001 vs H‐Exo; Figure 6D,H). Additionally, ARRDC3 knockdown significantly reduced OGD/R‐induced increases in MDA levels (*p* < 0.01 vs PBS; Figure 6I), intracellular Fe^2^^+^ accumulation (*p* < 0.001 vs PBS; Figure 6J), and concomitantly enhanced ATP production (*p* < 0.05 vs PBS; Figure 6K). Notably, ARRDC3 overexpression reversed these protective effects of H‐Exo, leading to increased MDA (*p* < 0.05 vs H‐Exo), elevated Fe^2^^+^ levels (*p* < 0.05 vs H‐Exo), and decreased ATP levels (*p* < 0.05 vs H‐Exo) (Figure 6I–K). These data reveal ARRDC3 as a critical upstream regulator of DRP1‐dependent mitochondrial dynamics, thereby mediating the anti‐ferroptotic effect of H‐Exo in OGD/R‐injured neurons.

### 2.6 H‐Exo Promotes Neuroprotection and Functional Recovery Through CRYAB–Mediated Inhibition of ARRDC3

**FIGURE 7:**
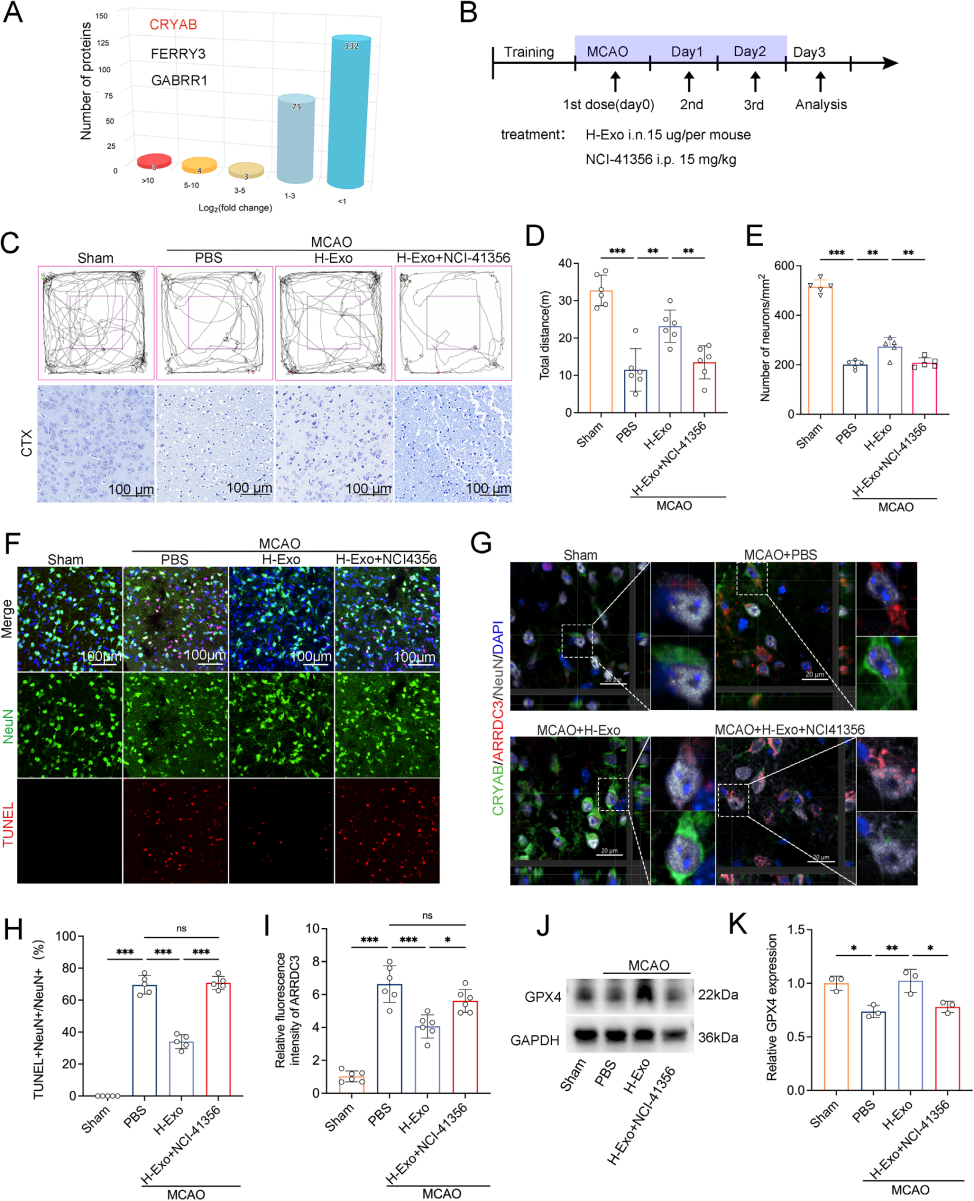
H‐Exo promotes neuroprotection and functional recovery through CRYAB–mediated inhibition of the ARRDC3. (A) Proteomic profiling of H‐Exo identified CRYAB as one of the most enriched proteins, with other significantly upregulated proteins including FERRY3 and GABRR1. (B) Schematic illustration of the experimental design. Mice were subjected to tMCAO followed by intranasal administration of H‐Exo (15 µg/mouse/day) administered with or without the NCI‐41356 (15 mg/kg, intraperitoneal injection) for three consecutive days. (C–E) Open field test assessing locomotor and anxiety‐like behaviors (C, top), and total distance traveled (D). *n* = 6 mice per group; Representative Nissl staining of the cortex (C, bottom) and quantification of surviving neurons per mm^2^. *n* = 5 mice per group. (F,H) Representative TUNEL/NeuN immunofluorescence staining (F) and corresponding quantification of TUNEL^+^NeuN^+^/total NeuN^+^ neurons (H). NeuN (green), TUNEL (red). *n* = 5 mice per group. (G,I) Representative immunofluorescence images showing CRYAB and ARRDC3 expression in the ischemic penumbra (G). H‐Exo treatment markedly reduced ARRDC3 expression, whereas co‐administration of NCI‐41356 partially reversed this effect, restoring ARRDC3 levels to those comparable to the PBS group. Quantitative analysis of ARRDC3 fluorescence intensity is shown in (I), confirming a significant reduction after H‐Exo treatment and a partial rescue by NCI‐41356. (J,K) Western blot analysis (J) and densitometric quantification (K) of GPX4 protein expression. *n* = 3 mice per group. Statistical analysis was performed using one‐way ANOVA. Data are presented as mean ± SD. ^*^ *p* < 0.05; ^**^ *p* < 0.01; ^***^ *p* < 0.001.

To elucidate the molecular mediators underlying the neuroprotective effects of H‐Exo, proteomic profiling was conducted. A total of 217 proteins were significantly upregulated in H‐Exo (Figure 7A). Most proteins exhibited moderate increases in abundance (log_2_FC < 3), while a subset showed strong enrichment (log_2_FC > 10), with CRYAB being among the most abundant and markedly upregulated proteins (Figure 7A). Gene set enrichment analysis (GSEA) revealed that these differentially expressed proteins were predominantly involved in pathways associated with acute‐phase response, oxidative stress regulation, apoptosis modulation and protein folding (Figure S3A), suggesting that H‐Exo participates in stress‐response and cell‐survival mechanisms.

Given the marked enrichment of CRYAB in H‐Exo, we next elucidated its functional role in vivo (Figure 7B). Mice were randomly assigned to four groups: Sham, MCAO + PBS, MCAO + H‐Exo, and MCAO + H‐Exo + NCI‐41356. Immediately after reperfusion, mice received intranasal administration of H‐Exo (15 µg per mouse per day), either alone or in combination with the CRYAB inhibitor NCI‐41356 (15 mg/kg, intraperitoneally) for three consecutive days (Figure 7B). Open‐field testing revealed that NCI‐41356 treatment partially attenuated the H‐Exo–mediated improvements in total distance traveled (Figure 7C,D), as well as the time spent (Figure S3B) and distance traveled in the central area (Figure S3C). Consistently, Nissl staining showed that NCI‐41356 treatment reversed the H‐Exo–induced reductions in neuronal loss and morphological damage in the ischemic cortex (Figure 7C,E), indicating that CRYAB activity is essential for the neuroprotective effects of H‐Exo. In line with the Nissl staining results, TUNEL analysis revealed that NCI‐41356 administration significantly reversed the H‐Exo–induced reduction in neuronal apoptosis (Figure 7F,H). Immunofluorescence staining of CRYAB and ARRDC3 showed that H‐Exo treatment markedly suppressed ARRDC3 expression in the ischemic penumbra (Figure 7G). Co‐administration of NCI‐41356 with H‐Exo reversed this effect, increasing ARRDC3 expression to levels similar to the PBS group. Quantitative analysis confirmed a significant reduction in ARRDC3 fluorescence intensity after H‐Exo treatment, which was partially rescued by NCI‐41356 (Figure 7I). Western blot analysis demonstrated that NCI‐41356 reduced H‐Exo‐induced GPX4 expression (Figure 7J,K), indicating that CRYAB activity is closely associated with the suppression of neuronal ferroptosis.

To confirm the specificity of the inhibitor, in vitro experiments were performed. Cells were divided into five groups: Control, OGD/R + PBS, OGD/R + H‐Exo, OGD/R + H‐Exo + NCI‐41356, and OGD/R + NCI‐41356 groups. EdU assays demonstrated that NCI‐41356 treatment notably weakened the H‐Exo–induced increase in cell proliferation (Figure S3D,E). Consistent with the in vivo findings, NCI‐41356 reduced the H‐Exo–mediated upregulation of GPX4 (Figure S3F,G) and reversed the decrease in MDA levels induced by H‐Exo (Figure S3H). Notably, NCI‐41356 treatment alone had no significant effect on cell proliferation or ferroptosis‐related markers under OGD/R conditions compared with the PBS group (Figure S3D–H). Collectively, these results demonstrate that H‐Exo provides potent neuroprotection against ischemic brain injury, and this protection is at least partially dependent on exosomal CRYAB activity, which suppresses ARRDC3‐mediated signaling, inhibiting neuronal apoptosis and ferroptosis, and promoting functional recovery.

### 2.7 Discussion

We previously demonstrated that intranasally delivered H‐Exo effectively targets microglia and suppresses neuroinflammation by regulating the HMGB1–TREM1–p38 MAPK pathway, thereby promoting neurological recovery after ischemic stroke [37]. However, the effects of H‐Exo on neuronal metabolic homeostasis and cell death pathways remain largely unclear. Given the critical role of mitochondrial dysfunction and ferroptosis in CIRI, we investigated whether H‐Exo alleviates oxidative damage and inhibits ferroptosis by maintaining mitochondrial homeostasis, thereby revealing a broader neuroprotective mechanism. Our findings demonstrate that H‐Exo provides potent neuroprotection against CIRI by modulating the CRYAB–ARRDC3–Drp1 pathway. Via nose‐to‐brain delivery, H‐Exo efficiently reduces mitochondrial hyperfission and oxidative stress, ultimately suppressing neuronal ferroptosis. Proteomic analysis combined with pharmacological validation identified CRYAB as a key upstream regulator of ARRDC3, and inhibition of CRYAB partially attenuated the therapeutic efficacy of H‐Exo, underscoring the central role of this signaling pathway. Collectively, these findings provide mechanistic insights into exosome‐mediated neuroprotection and highlight the translational potential of H‐Exo as a promising therapeutic strategy for ischemic stroke.

Our findings demonstrated that intranasally administered H‐Exo penetrated the blood–brain barrier (BBB) and accumulated within the ischemic penumbra, targeting over 80% of neurons, astrocytes, and microglia (Figure 1I–K). Consistently, previous studies have also shown that intranasally delivered human MSC–derived extracellular vesicles (MSC‐EVs) preferentially accumulate in brain tissue, exhibiting brain‐targeting capability and offering a promising strategy for treating neurological disorders [38]. Compared with the more heterogeneous and compositionally complex MSC‐EVs, the H‐Exo isolated in this study exhibited higher purity and well‐defined biological characteristics (Figure 1B–D), enabling more precise intercellular communication. Intranasal delivery allows therapeutic vesicles to reach the central nervous system directly via the olfactory and trigeminal nerve pathways, thereby safely and efficiently bypassing the BBB while minimizing peripheral exposure and systemic side effects [39, 40]. In our previous work, we demonstrated that intranasal administration of engineered M2pep‐ADSC‐Exos effectively inhibited ferroptosis in M2‐type microglia and significantly improved neurological recovery after ischemic stroke [41]. Collectively, these findings indicate that intranasal delivery of H‐Exo provides a safe, noninvasive, and efficient route for brain‐targeted and multi‐cellular exosome therapy in ischemic stroke.

Mechanistically, our findings demonstrate that H‐Exo inhibits ferroptosis by maintaining mitochondrial homeostasis. Previous studies have shown that hUMSC‐derived exosomes deliver circBBS2, which sponges miR‐494 and activates the SLC7A11/GPX4 pathway, thereby suppressing ferroptosis and promoting post‐stroke recovery [42]. This finding is consistent with our observation that H‐Exo attenuates ferroptosis‐associated neuronal injury. Beyond ferroptosis regulation, accumulating evidence also suggests that exosomes modulate mitochondrial dynamics to exert potent neuroprotection. For example, macrophage‐derived exosomes loaded with a Drp1/Fis1 inhibitory peptide P110 reduced astrocytic mitochondrial fragmentation, enhanced mitochondrial transfer to neurons, and alleviated ischemia–reperfusion injury [43]. Similarly, in a model of fatty liver disease, MSC‐Exos alleviated tissue injury by suppressing mitochondrial hyperfission and ferroptosis through the miR‐30b‐5p/Drp1 axis [44]. Beyond ischemic stroke, exosomes have also been reported to preserve mitochondrial function and energy homeostasis through regulating biogenesis, fusion–fission balance, axonal transport, and mitophagy, showing therapeutic promise in Alzheimer's and Parkinson's disease models [45, 46]. Collectively, these findings suggest that exosome‐based targeting of Drp1 to restore mitochondrial dynamics and suppress ferroptosis represents a promising and efficient therapeutic strategy for ischemic stroke, and may reflect a shared pathological mechanism across ischemic and neurodegenerative diseases. Building on these insights, although the interplay between mitochondrial dynamics and ferroptosis remains incompletely characterized, accumulating evidence suggests that mitochondrial dysregulation is a critical determinant of ferroptotic susceptibility. Aberrant mitochondrial fission and structural impairment have been implicated in initiating and amplifying ferroptosis [47, 48]. For instance, deletion of Drp1 effectively delays ferroptosis onset by preserving mitochondrial ultrastructure and reducing the accumulation of iron and reactive oxygen species (ROS), thereby exerting significant protective effects [49]. Conversely, another study further demonstrated that during ferroptosis induction, Drp1 phosphorylation and mitochondria translocation promote excessive mitochondrial fragmentation, lipid peroxidation, and oxidative damage [50]. Together, these findings identify Drp1 as a key mediator linking mitochondrial dysfunction and ferroptosis, forming a pathological feedback loop that accelerates cell death. In this context, our results indicate that H‐Exo may alleviate ischemia–reperfusion injury by modulating this pathway to preserve mitochondrial stability and suppress ferroptosis, establishing a multilevel neuroprotective mechanism.

In this study, we systematically investigated the role and mechanism of ARRDC3 in regulating mitochondrial dynamics and ferroptosis through gain‐ and loss‐of‐function experiments. Transcriptomic analysis revealed that *ARRDC3* was markedly upregulated in primary cortical neurons subjected to OGD/R, ranking among the top transcripts. ARRDC3, a membrane‐associated adaptor protein involved in endocytosis and intracellular trafficking, has been reported to suppress PPARγ activation and cell proliferation under stress conditions [51]. Its upregulation under hypoxia–ischemia may thus exacerbate neuronal vulnerability. Consistently, In preeclampsia models, ARRDC3 expression has been reported to be directly regulated by ischemia (hypoxia/reoxygenation), influencing the biological behavior of trophoblast cells under hypoxic conditions [52]. Moreover, ARRDC3 has been identified as a negative regulator of energy expenditure in metabolic regulation, with mouse studies demonstrating that its functional inhibition enhances β‐adrenergic signaling, thereby increasing energy consumption and improving metabolic health [53]. In line with this, our results showed that ARRDC3 overexpression in neurons decreased ATP production, confirming its detrimental effect on energy homeostasis. Bioinformatic analyses and related studies have identified ARRDC3 within the ferroptosis‐related gene network, suggesting a potential link to ischemia‐hypoxia‐related disorders [54, 55]. Functionally, this study for the first time verified the involvement of ARRDC3 in ferroptosis in neuronal hypoxia–reoxygenation models. ARRDC3 knockdown suppressed Drp1–mediated mitochondrial fission, preserved mitochondrial integrity, and reduced neuronal ferroptosis, whereas its overexpression promoted mitochondrial fragmentation and diminished the neuroprotective effects of exosomes, highlighting its key role in exosome‐mediated regulation of mitochondrial homeostasis and ferroptosis.

In exploring the protective mechanisms of exosomes, we further identified CRYAB (HSPB5) as a key functional protein in H‐Exo that plays a central role in regulating the ARRDC3–Drp1 signaling axis and mitochondrial dynamics. In our study, we found that H‐Exo suppress excessive mitochondrial fission and ferroptosis by targeting the ARRDC3–Drp1 pathway. Functional validation revealed that inhibition of CRYAB by NCI‐41356 partially reversed the neuroprotective effects of H‐Exo, supporting the critical upstream role of exosomal CRYAB in modulating ARRDC3‐mediated signaling. During CIRI, CRYAB is typically upregulated as a stress‐responsive factor and is considered an endogenous neuroprotective response to hypoxic injury. However, this upregulation is often transient and insufficient to counteract sustained oxidative stress and mitochondrial dysfunction. Previous studies have shown that Cryab^−^/^−^ mice exhibit larger infarct volumes and more severe neurological deficits following stroke, whereas exogenous administration of CRYAB markedly reduces infarct size and attenuates inflammation by suppressing pro‐inflammatory cytokines such as IL‐2, IL‐17, and TNF, while enhancing IL‐10 secretion, thereby improving neurological outcomes. Notably, therapeutic administration of CRYAB remains effective even when initiated 12 h after stroke onset, suggesting that reinforcement of this endogenous protective factor may extend the therapeutic window beyond conventional thrombolytic treatment [56]. Similarly, comparable protective effects have also been demonstrated in myocardial ischemia/reperfusion models, where enhanced CRYAB expression markedly inhibited ferroptosis, reduced infarct size, and improved cardiac function, further supporting its potent cytoprotective role in ischemic injury [57]. Altogether, these findings highlight CRYAB as a central stress‐responsive modulator that preserves mitochondrial homeostasis and inhibits ferroptosis, offering a mechanistic foundation for exosome‐based therapeutic strategies against ischemic injury.

### 2.8 Limitations

Although this study demonstrated that H‐Exo exert significant neuroprotective effects through the CRYAB–ARRDC3–Drp1 signaling pathway, several aspects warrant further exploration to strengthen and extend our conclusions. First, the mechanistic validation in this study was primarily conducted in vitro using neuronal models; therefore, additional in vivo investigations—particularly among different neuronal and glial subpopulations—will help confirm the physiological relevance of this pathway. Nevertheless, the strong consistency between cellular phenotypes and behavioral recovery supports the robustness of our findings. Second, although intranasal delivery ensures efficient brain targeting, the long‐term biodistribution and pharmacokinetic characteristics of exosomes remain incompletely understood. This limitation highlights the need for advanced imaging and quantitative tracking technologies to optimize exosome‐based therapeutic delivery. Finally, the precise molecular interface through which CRYAB regulates the ARRDC3–Drp1 pathway remains to be elucidated; clarifying this regulatory hierarchy will deepen our understanding of how exosomal cargoes fine‐tune mitochondrial dynamics and ferroptosis. Overall, these insights not only consolidate the mechanistic foundation of exosome‐mediated mitochondrial and ferroptosis regulation but also lay a solid framework for developing novel, clinically translatable exosome‐based therapeutic strategies.

## 3 Conclusion

This study reveals that H‐Exo provides neuroprotection against ischemic stroke by modulating the CRYAB–ARRDC3–Drp1 axis to inhibit ferroptosis and maintain mitochondrial homeostasis. We demonstrated that H‐Exo efficiently crosses the blood‐brain barrier, targets neurons, astrocytes, and microglia in the ischemic penumbra, and significantly alleviates neuronal injury caused by ischemia/reperfusion. Mechanistic analysis further identifies ARRDC3 as a key target of H‐Exo's anti‐ferroptotic effects, with CRYAB playing a crucial role in regulating this pathway. These findings provide new mechanistic insights into exosome‐based neuroprotection and highlight the potential of H‐Exo as a promising non‐invasive therapeutic strategy for ischemic stroke. Although H‐Exo exhibits significant protective effects in animal models, further studies are needed to explore its long‐term therapeutic efficacy and clinical translation, particularly regarding its precision‐targeted treatment and sustained therapeutic impact.

## 4 Experimental Methods

### 4.1 Ethics and Animals

Mice were purchased from Vital River (Beijing, China) and maintained in a controlled environment at the Animal Core Facility of the School of Basic Medical Sciences, Fudan University. All protocols were approved by the Institutional Animal Care and Use Committee of Fudan University (Approval No. 2023‐MHFY23JZS). Animals were provided with autoclaved water and regular chow ad libitum, and housed in groups of 5–6 per cage.

### 4.2 tMCAO Model Establishment

Randomly selected male C57BL/6 mice (8 weeks of age, weighing 23–26 g) were anesthetized via intraperitoneal injection of 1% pentobarbital. A 2 cm midline incision was made in the neck to expose the external, internal, and common carotid arteries (ECA, ICA, and CCA), which were subsequently isolated and ligated under sterile conditions. A 1.5 mm arteriotomy was created in the ECA to introduce a silicone‐coated intraluminal filament (MSMC21B120PK50, RWD, Shenzhen, China). Following distal ECA transection and ICA ligature removal, the filament was advanced retrograde into the ICA lumen. Advancement ceased upon encountering slight resistance, confirming middle cerebral artery (MCA) occlusion at the Circle of Willis. After 60 min ischemia, the filament was withdrawn, the ECA stump was permanently ligated with 8–0 polypropylene, and CCA flow was restored to initiate reperfusion. Multilayer closure employed 4–0 Vicryl sutures in fascial planes. Body temperature was strictly controlled at 37.0 ± 0.5°C with the aid of a heating pad. For the sham group, all surgical steps were replicated except for the insertion of the filament and vascular occlusion.

### 4.3 Cell culture and OGD/R

hUMSC were derived from human umbilical cords obtained from Guangzhou Saliai Stem Cell Science and Technology Co., Ltd. The cells cultured in DMEM/F12 (Gibco, Carlsbad, CA, USA) with 10% fetal bovine serum (FBS, Gibco, Carlsbad, CA, USA).

N2a cells (FH0424, FudanCell, Shanghai, China) were maintained in Dulbecco's modified Eagle's medium (DMEM, Gibco, Carlsbad, CA, USA) with 10% FBS in 37°C/5% CO2. Cells at passages 5 to 6 were used for subsequent experiments.

Primary cortical neurons were isolated from embryonic day 17 (E17) C57BL/6 mice (SPF Biotech, Beijing, China) under aseptic conditions. Fetal brains were dissected within 2 min post‐euthanasia and rinsed in ice‐cold PBS containing triple antibiotics (15240062, Thermo Fisher, USA). Under stereomicroscopic visualization, meninges and vascular membranes were meticulously removed. The isolated cortices were then transferred into 15 mL tubes containing PBS and underwent 3–4 cycles of controlled trituration using Pipett tips, alternating with 5 min sedimentation periods at unit gravity to achieve single‐cell dissociation. The cell suspension was then filtered through a 70 µm cell strainer (431751, Corning, Shenzhen, China) and centrifuged at 300 × g for 3 min. After counting, the cells were plated at a density of 2 × 10^5^ cells/cm^2^ on Matrigel‐coated 6‐well plates at 37°C/5% CO2. Initial adhesion was promoted by DMEM/F12 with 10% FBS. To induce neuronal differentiation, the medium was replaced 4–6 h later with Neurobasal Medium supplemented with B‐27 and GlutaMAX (Thermo Fisher). Neuronal identity and purity were validated at 7 days by immunocytochemical detection of MAP2/DAPI. After identification, the cells were used for subsequent experiments.

After adherence, the medium was replaced with glucose‐free MEM (Gibco, Carlsbad, CA, USA) without FBS for N2a cells, while primary neuronal cells were switched to glucose‐free Neurobasal medium (Gibco, Carlsbad, CA, USA). Cells were then incubated in a hypoxic chamber (95% N_2_, 5% CO_2_) for 4 h, followed by 24 h reoxygenation in complete medium under normoxic conditions. Control cells were maintained in normoxic conditions throughout the experiment.

### 4.4 Isolation, Characterization, Labeling, and Treatment of H‐Exo

After reaching 80%–90% confluence in complete DMEM/F12, cells were rinsed and maintained in FBS‐free medium for 48 h. The supernatant was cleared by sequential centrifugation at 300 × g for 10 min and 2000 x g (≈3400 rpm) for 20 min at 4°C, filtered (0.22 µm), and ultracentrifuged at 100 000 × g(≈35 000 rpm, SW41Ti rotor) for 70 min at 4°C. Pellets were washed once in PBS, re‐suspended, protein‐determined (BCA), and frozen at −80°C. For labeling, exosomes were mixed with PKH‐26 (Sigma), re‐isolated by ultracentrifugation at 100 000 g (≈35 000 rpm), twice washed in PBS, 0.22 µm‐filtered, and stored at −80°C until analysis.

For in vitro uptake, N2a cells were incubated with labeled exosomes (20 µg/mL) for 6 h, fixed, and visualized by confocal microscopy after DAPI counterstaining. For in vivo studies, tMCAO mice received either PBS or exosomes (15 µg) intranasally once daily for 3 days post‐reperfusion, administered in a supine position with alternating nostrils.

### 4.5 Pharmacological Inhibition of CRYAB

To investigate the functional role of CRYAB in exosome‐mediated neuroprotection, the small‐molecule inhibitor NCI‐41356 ((2S,3R)‐3‐methylglutamic acid hydrochloride salt, sc‐206572, Santa Cruz) was employed for pharmacological intervention. For in vivo experiments, mice received H‐Exo (15 µg/per mouse, intranasally) or NCI‐41356 (15 mg/kg/day, intraperitoneally) alone or in combination for three consecutive days after reperfusion. The vehicle group received an equivalent volume of saline. For in vitro studies, N2a cells were treated with NCI‐41356 (10 µm) together with exosomes immediately after OGD/R, while the control group received an equal volume of NaCl (vehicle).

### 4.6 Neurobehavioral Assessment

Neurological impairments were evaluated using the modified Neurological Severity Score (mNSS, scale 0–14), which comprehensively measures motor function, sensory responses, balance, and reflex activity. Tests included spontaneous activity (open field), limb symmetry (tail suspension), beam walking, and sensory response (vibrissae touch). Blinded observers scored animals; Mice with mNSS scores 6–12 was included, excluding those with severe complications (score > 12) or failed occlusion (score <6).

### 4.7 TTC Staining

Brains were snap‐frozen at ‐80°C (5 min), coronally sectioned into 5–6 slices with chilled blades, incubated in preheated 1% TTC (T8877, Sigma, USA) (15 min, dark), fixed with 4% PFA overnight at 4°C, and imaged the next day.

### 4.8 Nissl Staining

Nissl staining (Beyotime, Shanghai, China) was performed on 20 µm coronal brain sections collected 72 h after MCAO. Brain sections harvested 72 h post‐MCAO were fixed in 4% paraformaldehyde, rinsed with phosphate‐buffered saline (PBS), and incubated in 0.1% cresyl violet solution for 10 min. Subsequently, the sections were differentiated in 95% ethanol, dehydrated using a graded ethanol series, cleared with xylene, and coverslipped for microscopic examination.

### 4.9 CCK‐8 Viability Assay

N2a cells were seeded in 96‐well plates, exposed to the indicated treatments, and incubated with CCK‐8 reagent (FS1157, Fushen Bio, China) for 4 h. Optical density at 450 nm was measured on a SpectraMax M5 microplate reader (Molecular Devices, USA) to assess cell viability.

### 4.10 Lipid Peroxidation Assay (MDA Quantification)

Malondialdehyde content was quantified with a commercial kit (So131S, Beyotime, China) per the supplier's instructions. After lysis in chilled RIPA buffer, samples were clarified by centrifugation at 12 000 × g (≈13 400 rpm) for 10 min at 4°C. Supernatants were incubated with thiobarbituric acid at 95°C for 1 h, cooled, and absorbance at 532 nm was read on a microplate reader. MDA values were calculated from a standard curve and normalized to total protein (BCA assay).

### 4.11 FerroOrange Probe

The intracellular concentration of ferrous iron (Fe^2^^+^) was determined using a commercial iron detection kit (F374, Dojindo, Shanghai, China) in accordance with the manufacturer's guidelines. Cells were harvested, rinsed with ice‐cold PBS, and lysed in iron assay buffer. After centrifugation, the resulting supernatant was incubated with a Fe^2^^+^‐sensitive probe at 37°C for 60 min in the absence of light. Absorbance was recorded at 593 nm using a spectrophotometer, and iron levels were normalized to the total protein concentration.

### 4.12 Mitochondrial Reactive Oxygen Species Production

To assess mitochondrial ROS production, N2a cells from different groups were stained with 5 µm MitoSOX Red (Invitrogen) for 20 min at 37°C in the dark. Following staining, the cells were washed with warm DMEM and observed using a confocal microscope.

### 4.13 Detection of Mitochondrial Membrane Potential

JC‐1 staining (Beyotime, China) was applied to evaluate mitochondrial membrane potential (MMP) in N2a cells. Cells were incubated with the dye at 37°C for at least 20 min in darkness, followed by buffer washing. Fluorescence of J‐aggregates (red) and J‐monomers (green) was captured using a Nikon NSparc confocal microscope (Japan).

### 4.14 Immunofluorescence Staining

Samples were fixed with 4% PFA for 20 min at room‐temperature, followed by PBS washes. Blocking/permeabilization was performed with 5% donkey serum + 0.3% Triton X‐100 for 1 h. Primary antibodies were incubated overnight at 4°C. After PBS washes, secondary antibodies and DAPI were applied for 1 h. Following final washes, slides were mounted and stored at 4°C after 24–48 h air‐drying.

The following primary antibodies were used: anti‐IBA‐1 (rat, Abcam, ab283346, 1:1000), anti‐GFAP (mouse, Abcam, ab4648, 1:1000), anti‐DRP1 (rabbit, Proteintech, 12957‐1‐AP, 1:500), anti‐MAP2 (mouse, Sigma, m1406, 1:1000), anti‐NeuN (rabbit, Proteintech, 26975‐1‐AP, 1:500). Anti‐ARRDC3 (rabbit, Affinity, DF3517,1:100), anti‐CRYAB (mouse, Santa CRUZ, sc‐137129, 1:50), Anti‐NeuN (chicken, Invitrogen, PA5‐143567, 1:5000).

The following secondary antibodies were used: Alexa Fluro 488 Donkey anti‐mouse IgG (Jackson,1:1000), Alexa Fluro 594 Donkey anti‐mouse IgG (Jackson,1:1000), Alexa Fluro 594 Donkey anti‐rabbit IgG (Jackson,1:1000), Alexa Fluro 488 Donkey anti‐rabbit IgG (Jackson,1:1,000), Alexa Fluro 488 Donkey anti‐rat IgG (Jackson,1:1000), Alexa Fluro 488 Donkey anti‐chicken IgG (Jackson,1:1000), DAPI (Sigma, 1:2,000). Fluorescent images were acquired using a confocal microscope (Nikon NSparc, Japan) equipped with 60× and 100× oil‐immersion objectives (NA = 1.4). Images were captured at a resolution of 1024 × 1024 pixels. The resolution at 100× magnification was as follows: lateral resolution 100 nm and axial resolution 300 nm. All imaging parameters, including laser intensity and exposure time, were kept constant across samples to ensure comparability. 3D reconstruction and quantitative fluorescence image analysis were performed using Imaris software (version 9.6; Bitplane, Zürich, Switzerland).

### 4.15 EdU Essay

Following treatment, cell proliferation was evaluated using the Click‐iT EdU Alexa Fluor 488 Kit (Invitrogen, USA). N2a cells were incubated with 10 µm EdU (1:2000) for 2 h at 37°C in 5% CO_2_. Cells were then fixed with 4% PFA, permeabilized with 0.2% Triton X‐100, and reacted with the Click‐iT mixture for 30 min in the dark. After DAPI (1:2000) staining, slides were air‐dried and imaged using confocal microscopy.

### 4.16 Open Field Test

Mice were individually introduced into a 40 × 40 × 40 cm open‐field chamber under low‐light conditions (50 lux) following a 1 h habituation period in the testing room. They were allowed to explore freely for 5 min. Locomotor behaviors, including total distance moved and time spent in the central zone, were recorded using the ANY‐maze automated tracking system. Between sessions, the arena was disinfected with 70% ethanol. Mice exhibiting motor dysfunction were excluded from analysis.

### 4.17 Western Blot Analysis

Cells and tissues were homogenized in RIPA lysis buffer supplemented with protease and phosphatase inhibitors, then kept on ice for 30 min. Protein content was measured using a BCA kit, and 25 µg of each sample was loaded for 12% SDS‐PAGE, followed by transfer to PVDF membranes (Millipore, USA). Membranes were blocked in 5% non‐fat milk (in TBST with 0.1% Tween‐20) at room‐temperature for 1 h and incubated overnight at 4°C with primary antibodies. After washing in TBST, species‐specific HRP‐conjugated secondary antibodies were added and incubated for another hour. Signal detection was performed using an ECL substrate kit (Millipore), and images were acquired with a Tanon‐4600 system (Tanon, Shanghai, China).

The following primary antibodies were used: anti‐DRP1 (rabbit, Proteintech, 12957‐1‐AP, 1:5000), anti‐GPX4 (rabbit, HUABIO, ET1706‐45, 1:10 000), anti‐COX4 (rabbit, Beyotime, AG8011, 1:1000), anti‐GAPDH (ET1702‐66, 1:50 000), anti‐ARRDC3 (rabbit, DF3517, Affinity, 1:200). Anti‐TSG101(ab125011, Abcam), anti‐Alix (ab117600, Abcam). The following secondary antibodies were used: HRP‐conjugated goat anti‐rabbit IgG (HUABIO, HA1001, 1:50 000) and HRP‐conjugated goat anti‐mouse IgG (HUABIO, HA1006, 1:50 000).

### 4.18 In Vivo Fluorescence Imaging

For the sham group, mice received 6 µL of HUMSC‐derived exosomes that had been fluorescently labeled with DiR (MCE, NJ, USA) and purified via ultracentrifugation at 120 000 x g (≈36,000 rpm, SW41Ti rotor) for 70 min. The exosome suspension was administered dropwise into alternating nostrils (1 µL per nostril every 2 min). After 12 h, the animals were euthanized, their brains were rapidly removed, dried, and placed on a non‐reflective black surface for ex vivo near‐infrared imaging using the IVIS Spectrum system (excitation 745 nm).

### 4.19 ATP Assay

ATP levels were measured using a bioluminescent assay kit (HY‐K0314, MCE, USA). Briefly, 100 µL detection reagent was added to 96‐well opaque plates and equilibrated for 5 min. Subsequently, 20 µL of sample or standard was introduced, gently mixed, and luminescence was recorded immediately (SpectraMax M5, Molecular Devices, USA). Values were calculated using a standard curve and normalized to total protein (BCA assay).

### 4.20 RNA‐seq and Data Analysis

RNA sequencing was performed on neurons from three experimental groups, each with three biological replicates (*n* = 3 per group). Total RNA was extracted using standard protocols, and RNA integrity was assessed with an Agilent 2100 bioanalyzer. mRNA was obtained either by polyA selection with Oligo(dT) beads or by rRNA depletion, followed by random fragmentation in NEB Fragmentation Buffer and library construction (NEB, USA). Libraries were quantified, insert sizes were checked, and qRT‐PCR was used to determine effective concentrations before sequencing on an Illumina platform with PE150 configuration.

Adapter and low‐quality sequences were trimmed from raw reads with fastp (v0.20.0). Clean reads were mapped to the mouse reference genome (mm10, UCSC) using HISAT2, and the resulting alignments were sorted and indexed by SAMtools. Transcripts were reconstructed with StringTie, and gene‐level abundances were estimated as FPKM via FeatureCounts. Differential expression analysis was performed with DESeq2, adopting cut‐offs of adjusted *P* < 0.05 and |log_2_FC| > 0.58.

### 4.21 TUNEL Assay

Following permeabilization and blocking, sections were rinsed in PBS and subjected to TUNEL staining (K1134, APExBIO, USA) per the manufacturer's instructions. Standard immunofluorescence staining was then performed using specific antibodies.

### 4.22 Mitochondrial Isolation

Mitochondrial proteins were isolated using a commercial extraction kit (C3601, Beyotime, China). Briefly, ∼2 × 10^7^ cells were collected, resuspended in isolation buffer, and kept on ice for 15 min. After homogenization to disrupt ∼50% of the cells, the mixture was centrifuged at 600 × g for 10 min at 4°C to remove nuclei and debris. The supernatant was spun again at 11 000 × g, 4°C, 10 min to pellet mitochondria. The resulting supernatant was further cleared at 12 000 × g, 4°C, 10 min to yield the cytosolic fraction. Mitochondrial pellets were washed, lysed in fresh buffer on ice for 30 min, and clarified at 12 000 × g, 4°C, 10 min; the final supernatant was collected as the mitochondrial protein fraction.

### 4.23 Quantitative Real‐Time PCR (qRT‐PCR)

Total RNA was isolated with the EZ‐press kit (B0004D, EZB, USA). Concentration and purity were evaluated on a NanoDrop spectrophotometer; only samples exhibiting A260/A280 values of 1.8–2.0 were retained. First‐strand cDNA was generated using a reverse‐transcription kit (A0010CGQ, EZB, USA). qPCR runs were carried out on a StepOnePlus instrument (Thermo Fisher Scientific) with 2 × SYBR Green master mix (A0012‐R1, EZB, USA). Relative gene expression was calculated by the 2^–ΔΔCt method, normalized to Gapdh. Primer pairs are provided in Supplementary Table S1.

### 4.24 Si‐ARRDC3 and ARRDC3 Overexpression

For RNA interference‐based gene knockdown experiments, CALNP RNAi transfection reagent (DN001, D‐Nano Therapeutics) was used to transiently transfect small interfering RNA targeting ARRDC3 (si‐ARRDC3: sense: GGACCACUGUUUGCUUAUA, antisense: UAUAAGCAAACAGUGGUCC into N2a cells for 48 h. For gene overexpression, ARRDC3 was integrated into the N2a genome using lentiviral transduction (MOI = 15) with a construct obtained from Genomeditech (Shanghai, China).

### 4.25 Co‐Immunoprecipitation (Co‐IP)

Co‐immunoprecipitation was performed using the BeyoMag Protein A+G Magnetic Beads Kit (Beyotime, P2108) according to the manufacturer's instructions. Cell samples were lysed on ice with pre‐cooled lysis buffer supplemented with protease and phosphatase inhibitor cocktails. After lysis for 30 min at 4°C with gentle rotation, lysates were centrifuged at 12 000 × g for 15 min, and the supernatant was collected as total protein. Pre‐washed BeyoMag Protein A+G magnetic beads (30–40 µL) were incubated separately with the primary antibody against the target protein or an equivalent amount of species‐matched IgG (negative control) at 4°C for 1 h with gentle rotation to allow sufficient antibody coupling to the bead surface. A portion of the total protein (5%–10%) was reserved as an input control. The antibody‐conjugated beads were then incubated with an equal amount of total protein at 4°C overnight to allow antigen–antibody complex formation. After incubation, the beads were collected using a magnetic stand and washed four to five times with cold IP lysis buffer to remove nonspecific binding. Bound proteins were eluted by adding 1 × SDS loading buffer and boiling at 95°C for 5 min. The eluted proteins were analyzed by SDS‐PAGE followed by immunoblotting.

The primary antibodies used included anti‐DRP1 (for IP and WB; rabbit, Proteintech, 12957‐1‐AP), anti‐IgG (for IP; rabbit, Proteintech, 30000‐0‐AP), and anti‐ARRDC3 (for WB; rabbit, Affinity, DF3517, 1:200). An HRP‐conjugated Mouse Anti‐Rabbit IgG secondary antibody (Abmart, M21006, 1:3,000), optimized to minimize heavy‐chain interference, was used for Western blot detection.

### 4.26 Proteomic Profiling and Analysis

To identify bioactive cargo in H‐Exo, proteomic profiling was performed using label‐free quantitative mass spectrometry, with data derived from our previous study [37].

### 4.27 Statistical Analysis

Statistical analysis was performed using GraphPad Prism 10.0 (GraphPad Software, USA). For normally distributed data, comparisons between two groups were conducted using Student's *t*‐test. One‐way or two‐way ANOVA was applied for multi‐group comparisons. A *p*‐value < 0.05 was considered significant.

## Funding

This work was supported by the National Natural Science Foundation of China (Grant Nos. 82173646, 82373703 and 82501570), the Public Health Discipline Construction Project of Shanghai Minhang District Health Commission (Grant No. MGWXK2023‐04), and the China Postdoctoral Science Foundation (Grant No. 2024M750535).

## Conflicts of Interest

The authors declare no conflict of interest.

## Supporting information




**Supporting File 1**: adhm70791‐sup‐0001‐SuppMat.pdf.

**Supporting File 2**: adhm70791‐sup‐0002‐DataFile.pdf.

## Data Availability

The data that support the findings of this study are available from the corresponding author upon reasonable request.

## References

[1] S. S. Martin , A. W. Aday , N. B. Allen , et al., “2025 Heart Disease and Stroke Statistics: A Report of Us and Global Data From the American Heart Association,” Circulation 151, no. 8 (2025): e41–e660, 10.1161/cir.0000000000001303. 39866113 PMC12256702

[2] S. K. Feske , “Ischemic Stroke,” The American Journal of Medicine 134, no. 12 (2021): 1457–1464, 10.1016/j.amjmed.2021.07.027. 34454905

[3] M. C. Antonio , P. Sotirios , G. Stefano , et al., “Strategies of Cerebral Protection and Neurologic Dysfunctions After Circulatory Arrest: Back to the Future?,” Vessel Plus 7 (2023): 18, 10.20517/2574-1209.2023.42.

[4] Y. Xiong , B. C. V. Campbell , L. H. Schwamm , et al., “Tenecteplase for Ischemic Stroke at 4.5 to 24 H Without Thrombectomy,” New England Journal of Medicine 391, no. 3 (2024): 203–212, 10.1056/NEJMoa2402980. 38884324

[5] P. Jolugbo and R. A. S. Ariëns , “Thrombus Composition and Efficacy of Thrombolysis and Thrombectomy in Acute Ischemic Stroke,” Stroke; A Journal of Cerebral Circulation 52, no. 3 (2021): 1131–1142, 10.1161/strokeaha.120.032810. PMC7610448 33563020

[6] C. Orset , K. Arkelius , A. Anfray , K. Warfvinge , D. Vivien , and S. Ansar , “Combination Treatment With U0126 and Rt‐Pa Prevents Adverse Effects of the Delayed Rt‐Pa Treatment After Acute Ischemic Stroke,” Scientific Reports 11, no. 1 (2021): 11993, 10.1038/s41598-021-91469-9. 34099834 PMC8184783

[7] W. S. Toh , R. C. Lai , B. Zhang , and S. K. Lim , “Msc Exosome Works Through a Protein‐Based Mechanism of Action,” Biochemical Society Transactions 46, no. 4 (2018): 843–853, 10.1042/bst20180079. 29986939 PMC6103455

[8] M. Broadwin , G. Aghagoli , S. A. Sabe , et al., “Extracellular Vesicle Treatment Partially Reverts Epigenetic Alterations in Chronically Ischemic Porcine Myocardium,” Vessel Plus 7 (2023): 25, 10.20517/2574-1209.2023.103. 37982029 PMC10656099

[9] T. Li , M. Xia , Y. Gao , Y. Chen , and Y. Xu , “Human Umbilical Cord Mesenchymal Stem Cells: An Overview of Their Potential in Cell‐Based Therapy,” Expert Opinion on Biological Therapy 15, no. 9 (2015): 1293–1306, 10.1517/14712598.2015.1051528. 26067213

[10] J. W. Jung , M. Kwon , J. C. Choi , et al., “Familial Occurrence of Pulmonary Embolism After Intravenous, Adipose Tissue‐Derived Stem Cell Therapy,” Yonsei Medical Journal 54, no. 5 (2013): 1293–1296, 10.3349/ymj.2013.54.5.1293. 23918585 PMC3743204

[11] C. M. Xu , M. Broadwin , P. Faherty , et al., “Lack of Cardiac Benefit After Intramyocardial or Intravenous Injection of Mesenchymal Stem Cell‐Derived Extracellular Vesicles Supports the Need for Optimized Cardiac Delivery,” Vessel Plus 7 (2023): 33, 10.20517/2574-1209.2023.98. 38812773 PMC11136491

[12] Y. Yaghoubi , A. Movassaghpour , M. Zamani , M. Talebi , A. Mehdizadeh , and M. Yousefi , “Human Umbilical Cord Mesenchymal Stem Cells Derived‐Exosomes in Diseases Treatment,” Life Sciences 233 (2019): 116733, 10.1016/j.lfs.2019.116733. 31394127

[13] M. Zhang , Q. Liu , H. Meng , et al., “Ischemia‐Reperfusion Injury: Molecular Mechanisms and Therapeutic Targets,” Signal Transduction and Targeted Therapy 9, no. 1 (2024): 12, 10.1038/s41392-023-01688-x. 38185705 PMC10772178

[14] A. Jurcau and A. Simion , “Neuroinflammation in Cerebral Ischemia and Ischemia/Reperfusion Injuries: From Pathophysiology to Therapeutic Strategies,” International Journal of Molecular Sciences 23, no. 1 (2021): 14, 10.3390/ijms23010014. 35008440 PMC8744548

[15] R. She , D. Liu , J. Liao , G. Wang , J. Ge , and Z. Mei , “Mitochondrial Dysfunctions Induce Panoptosis and Ferroptosis in Cerebral Ischemia/Reperfusion Injury: From Pathology to Therapeutic Potential,” Frontiers in Cellular Neuroscience 17 (2023): 1191629, 10.3389/fncel.2023.1191629. 37293623 PMC10244524

[16] M. Wu , X. Gu , and Z. Ma , “Mitochondrial Quality Control in Cerebral Ischemia–Reperfusion Injury,” Molecular Neurobiology 58, no. 10 (2021): 5253–5271, 10.1007/s12035-021-02494-8. 34275087

[17] M. Adebayo , S. Singh , A. P. Singh , and S. Dasgupta , “Mitochondrial Fusion and Fission: The Fine‐Tune Balance for Cellular Homeostasis,” The FASEB Journal 35, no. 6 (2021): 21620, 10.1096/fj.202100067R. PMC8415099 34048084

[18] W. Chen , H. Zhao , and Y. Li , “Mitochondrial Dynamics in Health and Disease: Mechanisms and Potential Targets,” Signal Transduction and Targeted Therapy 8, no. 1 (2023): 333, 10.1038/s41392-023-01547-9. 37669960 PMC10480456

[19] Y. J. Yuan , T. Chen , Y. L. Yang , H. N. Han , and L. M. Xu , “E2F1/CDK5/DRP1 Axis Mediates Microglial Mitochondrial Division and Autophagy in the Pathogenesis of Cerebral Ischemia‐Reperfusion Injury,” Clinical and Translational Medicine 15, no. 2 (2025): 70197, 10.1002/ctm2.70197. PMC11836619 39968698

[20] X. Zeng , Y.‐D. Zhang , R.‐Y. Ma , et al., “Activated Drp1 Regulates P62‐Mediated Autophagic Flux and Aggravates Inflammation in Cerebral Ischemia‐Reperfusion Via the Ros‐Rip1/Rip3‐Exosome Axis,” Military Medical Research 9, no. 1 (2022): 25, 10.1186/s40779-022-00383-2. 35624495 PMC9137164

[21] A. R. Anzell , G. M. Fogo , Z. Gurm , et al., “Mitochondrial Fission and Mitophagy Are Independent Mechanisms Regulating Ischemia/Reperfusion Injury in Primary Neurons,” Cell Death & Disease 12, no. 5 (2021): 475, 10.1038/s41419-021-03752-2. 33980811 PMC8115279

[22] S. J. Dixon , K. M. Lemberg , M. R. Lamprecht , et al., “Ferroptosis: An Iron‐Dependent Form of Nonapoptotic Cell Death,” Cell 149, no. 5 (2012): 1060–1072, 10.1016/j.cell.2012.03.042. 22632970 PMC3367386

[23] Y. Wang , H. Niu , L. Li , et al., “Anti‐Chac1 Exosomes for Nose‐to‐Brain Delivery of Mir‐760‐3p in Cerebral Ischemia/Reperfusion Injury Mice Inhibiting Neuron Ferroptosis,” Journal of Nanobiotechnology 21, no. 1 (2023): 109, 10.1186/s12951-023-01862-x. 36967397 PMC10041751

[24] X. Jiang , B. R. Stockwell , and M. Conrad , “Ferroptosis: Mechanisms, Biology and Role in Disease,” Nature Reviews Molecular Cell Biology 22, no. 4 (2021): 266–282, 10.1038/s41580-020-00324-8. 33495651 PMC8142022

[25] B. Zhou , J. Liu , R. Kang , D. J. Klionsky , G. Kroemer , and D. Tang , “Ferroptosis Is a Type of Autophagy‐Dependent Cell Death,” Seminars in Cancer Biology 66 (2020): 89–100, 10.1016/j.semcancer.2019.03.002. 30880243

[26] B. Wang , Y. Wang , J. Zhang , et al., “Ros‐Induced Lipid Peroxidation Modulates Cell Death Outcome: Mechanisms Behind Apoptosis, Autophagy, and Ferroptosis,” Archives of Toxicology 97, no. 6 (2023): 1439–1451, 10.1007/s00204-023-03476-6. 37127681

[27] Z. Miao , W. Tian , Y. Ye , et al., “Hsp90 Induces Acsl4‐Dependent Glioma Ferroptosis Via Dephosphorylating Ser637 at Drp1,” Cell Death & Disease 13, no. 6 (2022): 548, 10.1038/s41419-022-04997-1. 35697672 PMC9192632

[28] S. Hao , H. Huang , R. Y. Ma , X. Zeng , and C. Y. Duan , “Multifaceted Functions of Drp1 in Hypoxia/Ischemia‐Induced Mitochondrial Quality Imbalance: From Regulatory Mechanism to Targeted Therapeutic Strategy,” Military Medical Research 10, no. 1 (2023): 46, 10.1186/s40779-023-00482-8. 37833768 PMC10571487

[29] D. Yu , Y. Mei , L. Wang , et al., “Nano‐Seq Analysis Reveals Different Functional Tendency Between Exosomes and Microvesicles Derived From Humsc,” Stem Cell Research & Therapy 14, no. 1 (2023): 272, 10.1186/s13287-023-03491-5. 37749641 PMC10521478

[30] Y. U.‐S. Fu , C.‐C. Yeh , P.‐M. Chu , W.‐H. Chang , M.‐Y. A. Lin , and Y.‐Y. Lin , “Xenograft of Human Umbilical Mesenchymal Stem Cells Promotes Recovery From Chronic Ischemic Stroke in Rats,” International Journal of Molecular Sciences 23, no. 6 (2022): 3149, 10.3390/ijms23063149. 35328574 PMC8953545

[31] H. Abbaszadeh , F. Ghorbani , M. Derakhshani , A. Movassaghpour , and M. Yousefi , “Human Umbilical Cord Mesenchymal Stem Cell‐Derived Extracellular Vesicles: A Novel Therapeutic Paradigm,” Journal of Cellular Physiology 235, no. 2 (2020): 706–717, 10.1002/jcp.29004. 31254289

[32] J. I. Che , H. Wang , J. Dong , et al., “Human Umbilical Cord Mesenchymal Stem Cell–Derived Exosomes Attenuate Neuroinflammation and Oxidative Stress Through the NRF2 / NF‐κB / NLRP3 Pathway,” CNS Neuroscience & Therapeutics 30, no. 3 (2024): 14454, 10.1111/cns.14454. PMC10916441 37697971

[33] Z. Hu , Y. A. Yuan , X. Zhang , et al., “Human Umbilical Cord Mesenchymal Stem Cell‐Derived Exosomes Attenuate Oxygen‐Glucose Deprivation/Reperfusion‐Induced Microglial Pyroptosis by Promoting FOXO3a‐Dependent Mitophagy,” Oxidative Medicine and Cellular Longevity 2021 (2021): 6219715, 10.1155/2021/6219715. 34765084 PMC8577931

[34] W.‐J. Hu , H. Wei , L. I.‐L. I. Cai , et al., “Magnetic Targeting Enhances the Neuroprotective Function of Human Mesenchymal Stem Cell‐Derived Iron Oxide Exosomes by Delivering Mir‐1228‐5p,” Journal of Nanobiotechnology 22, no. 1 (2024): 665, 10.1186/s12951-024-02941-3. 39468528 PMC11514807

[35] X. Chen , T. Yang , Y. Zhou , Z. Mei , and W. Zhang , “Astragaloside IV Combined With Ligustrazine Ameliorates Abnormal Mitochondrial Dynamics via Drp1 SUMO/DeSUMOylation in Cerebral Ischemia–Reperfusion Injury,” CNS Neuroscience & Therapeutics 30, no. 4 (2024): 14725, 10.1111/cns.14725. PMC11016344 38615367

[36] H. Wedegaertner , W. A. Pan , C. C. Gonzalez , D. J. Gonzalez , and J. Trejo , “The α‐Arrestin Arrdc3 Is an Emerging Multifunctional Adaptor Protein in Cancer,” Antioxidants & Redox Signaling 36, no. 13‐15 (2022): 1066–1079, 10.1089/ars.2021.0193. 34465145 PMC9127825

[37] Z. Zhang , R. Ji , Z. Liu , et al., “Humsc‐Exosomes Suppress Trem1‐P38 Mapk Signaling Via Hmgb1‐Dependent Mechanisms to Reprogram Microglial Function and Promote Neuroprotection in Ischemic Stroke,” Journal of Nanobiotechnology 23, no. 1 (2025): 572, 10.1186/s12951-025-03652-z. 40830888 PMC12363081

[38] A. M. Tolomeo , G. Zuccolotto , R. Malvicini , et al., “Biodistribution of Intratracheal, Intranasal, and Intravenous Injections of Human Mesenchymal Stromal Cell‐Derived Extracellular Vesicles in a Mouse Model for Drug Delivery Studies,” Pharmaceutics 15, no. 2 (2023): 548, 10.3390/pharmaceutics15020548. 36839873 PMC9964290

[39] J. Koo , C. Lim , and K. T. Oh , “Recent Advances in Intranasal Administration for Brain‐Targeting Delivery: A Comprehensive Review of Lipid‐Based Nanoparticles and Stimuli‐Responsive Gel Formulations,” International Journal of Nanomedicine 19 (2024): 1767–1807, 10.2147/ijn.S439181. 38414526 PMC10898487

[40] S. Gandhi , D. H. Shastri , J. Shah , A. B. Nair , and S. Jacob , “Nasal Delivery to the Brain: Harnessing Nanoparticles for Effective Drug Transport,” Pharmaceutics 16, no. 4 (2024): 481, 10.3390/pharmaceutics16040481. 38675142 PMC11055100

[41] Y. Wang , Z. Liu , L. Li , et al., “Anti‐Ferroptosis Exosomes Engineered for Targeting M2 Microglia to Improve Neurological Function in Ischemic Stroke,” Journal of Nanobiotechnology 22, no. 1 (2024): 291, 10.1186/s12951-024-02560-y. 38802919 PMC11129432

[42] T. Hong , T. Zhao , W. He , et al., “Exosomal circBBS2 Inhibits Ferroptosis by Targeting miR‐494 to Activate SLC7A11 Signaling in Ischemic Stroke,” The FASEB Journal 37, no. 9 (2023): 23152, 10.1096/fj.202300317RRR. 37603538

[43] W. Liu , C. Su , Y. Qi , J. Liang , L. Zhao , and Y. Shi , “Brain‐Targeted Heptapeptide‐Loaded Exosomes Attenuated Ischemia–Reperfusion Injury by Promoting the Transfer of Healthy Mitochondria From Astrocytes to Neurons,” Journal of Nanobiotechnology 20, no. 1 (2022): 242, 10.1186/s12951-022-01425-6. 35606779 PMC9125840

[44] Y. Chen , F. Yang , Y. Wang , et al., “Mesenchymal Stem Cell‐Derived Small Extracellular Vesicles Reduced Hepatic Lipid Accumulation in Masld by Suppressing Mitochondrial Fission,” Stem Cell Research & Therapy 16, no. 1 (2025): 116, 10.1186/s13287-025-04228-2. 40045380 PMC11884000

[45] B. O. Li , Y. Chen , Y. Zhou , et al., “Neural Stem Cell‐Derived Exosomes Promote Mitochondrial Biogenesis and Restore Abnormal Protein Distribution in a Mouse Model of Alzheimer's Disease,” Neural Regeneration Research 19, no. 7 (2024): 1593–1601, 10.4103/1673-5374.385839. 38051904 PMC10883488

[46] Y. H. Jung , H. Y. Jo , D. H. Kim , et al., “Exosome‐Mediated Mitochondrial Regulation: A Promising Therapeutic Tool for Alzheimer's Disease and Parkinson's Disease,” International Journal of Nanomedicine 20 (2025): 4903–4917, 10.2147/ijn.S513816. 40259919 PMC12011032

[47] J. Li , Y.‐C. Jia , Y.‐X. Ding , J. Bai , F. Cao , and F. Li , “The Crosstalk Between Ferroptosis and Mitochondrial Dynamic Regulatory Networks,” International Journal of Biological Sciences 19, no. 9 (2023): 2756–2771, 10.7150/ijbs.83348. 37324946 PMC10266069

[48] Z. Wang , S. Tang , L. Cai , et al., “Drp1 Inhibition‐Mediated Mitochondrial Elongation Abolishes Cancer Stemness, Enhances Glutaminolysis, and Drives Ferroptosis in Oral Squamous Cell Carcinoma,” British Journal of Cancer 130, no. 11 (2024): 1744–1757, 10.1038/s41416-024-02670-2. 38582810 PMC11130175

[49] S. Tang , A. Fuß , Z. Fattahi , and C. Culmsee , “Drp1 Depletion Protects Against Ferroptotic Cell Death by Preserving Mitochondrial Integrity and Redox Homeostasis,” Cell Death & Disease 15, no. 8 (2024): 626, 10.1038/s41419-024-07015-8. 39191736 PMC11350090

[50] L. Pedrera , L. Prieto Clemente , A. Dahlhaus , et al., “Ferroptosis Triggers Mitochondrial Fragmentation Via Drp1 Activation,” Cell Death & Disease 16, no. 1 (2025): 40, 10.1038/s41419-024-07312-2. 39863602 PMC11762985

[51] S.‐I. Oka , H. Masutani , W. Liu , et al., “Thioredoxin‐Binding Protein‐2‐Like Inducible Membrane Protein Is a Novel Vitamin D3 and Peroxisome Proliferator‐Activated Receptor (PPAR)γ Ligand Target Protein that Regulates PPARγ Signaling,” Endocrinology 147, no. 2 (2006): 733–743, 10.1210/en.2005-0679. 16269462

[52] D. Lei , N. Deng , S. Wang , J. Huang , and C. Fan , “Upregulated Arrdc3 Limits Trophoblast Cell Invasion and Tube Formation and Is Associated With Preeclampsia,” Placenta 89 (2020): 10–19, 10.1016/j.placenta.2019.10.009. 31665660

[53] P. Patwari , V. Emilsson , E. E. Schadt , et al., “The Arrestin Domain‐Containing 3 Protein Regulates Body Mass and Energy Expenditure,” Cell Metabolism 14 (2011): 671–683, 10.1016/j.cmet.2011.08.011. 21982743 PMC3216113

[54] S. Guo , Z. Gong , X. Sun , et al., “Consensus Clustering Analysis Identifies Ferroptosis‐Related Patient Clusters and Predictive Signature Construction Based on Ferroptosis‐Related Genes in Ischemic Cardiomyopathy,” Journal of Inflammation Research 17 (2024): 6797–6814, 10.2147/jir.S475645. 39372582 PMC11451430

[55] J. Lin , S. Lin , Y. Zhang , and W. Liu , “Identification of Ferroptosis‐Related Potential Biomarkers and Immunocyte Characteristics in Chronic Thromboembolic Pulmonary Hypertension Via Bioinformatics Analysis,” BMC Cardiovascular Disorders 23, no. 1 (2023): 504, 10.1186/s12872-023-03511-5. 37821869 PMC10566044

[56] A. Arac , S. E. Brownell , J. B. Rothbard , et al., “Systemic Augmentation of αB‐Crystallin Provides Therapeutic Benefit Twelve Hours Post‐Stroke Onset via Immune Modulation,” Proceedings of the National Academy of Sciences 108, no. 32 (2011): 13287–13292, 10.1073/pnas.1107368108. PMC3156222 21828004

[57] A. Wu , C. Zhong , X. Song , et al., “The Activation of Lbh‐Cryab Signaling Promotes Cardiac Protection Against I/R Injury by Inhibiting Apoptosis and Ferroptosis,” iScience 27, no. 5 (2024): 109510, 10.1016/j.isci.2024.109510. 38660406 PMC11039335

