# Supplementary material for: Targeting the ARRDC3–DRP1 Axis via hUMSC‐Derived Exosomal CRYAB for Neuroprotection in Cerebral Ischemia/Reperfusion Injury
Source: Adv Healthc Mater. 2026 Jan 19;15(14):e03803. doi: 10.1002/adhm.202503803 (PMC13068360; doi:10.1002/adhm.202503803)
Supplement: Supplementary file 1 — Supporting File 1: adhm70791‐sup‐0001‐SuppMat.pdf. [file ADHM-15-0-s001.docx]

Supporting Information

**Targeting the ARRDC3–DRP1 Axis via hUMSC-Derived Exosomal CRYAB for Neuroprotection in Cerebral Ischemia/Reperfusion Injury**

Rong ji^1^, Zengyu Zhang^1^, Zhuohang Liu^2^, Kaicheng Yang^1^, Xueyu Mao^1^, Min Chu^3^, Yong Wang^4*^, Jing Zhao^1,5*^

^1^Department of Neurology, Minhang Hospital, Fudan University, Shanghai, China;

^2^Department of Rehabilitation Medicine, Zhongshan Hospital, Fudan University, Shanghai, China;

^3^Department of Geriatrics, Shanghai Geriatric Medical Center, Shanghai, China;

^4^Department of Neurology, Zhongshan Hospital, Fudan University, Shanghai, China;

^5^Institute of Healthy Yangtze River Delta, Shanghai Jiao Tong University, Shanghai, China

Correspondence: Jing Zhao ([zhao_jing@fudan.edu.cn](mailto:zhao_jing@fudan.edu.cn)); Yong Wang ([yong_wang@fudan.edu.cn](mailto:yong_wang@fudan.edu.cn))

Rong ji and Zengyu Zhang contributed equally to this work.

Table S1. List of primer sequences used for qRT-PCR analysis.

| **Gene** | **Sequence** |
| --- | --- |
| *Arrdc3-F* | ATGGTGCTGGGAAAGGTAAAG |
| *Arrdc3-R* | CGCTAGAATACACGGGGACATTA |
| *Hspb1-F* | ATCCCCTGAGGGCACACTTA |
| *Hspb1-R* | GGAATGGTGATCTCCGCTGAC |
| *Timp1-F* | GCAACTCGGACCTGGTCATAA |
| *Timp1-R* | CGGCCCGTGATGAGAAACT |
| *Cav1-F* | ATGTCTGGGGGCAAATACGTG |
| *Cav1-R* | CGCGTCATACACTTGCTTCT |
| *Ndrg1-F* | ATGTCCCGAGAGCTACATGAC |
| *Ndrg1-R* | CCTGCTCCTGAACATCGAACT |
| *H19-F* | GAACAGAAGCATTCTAGGCTGG |
| *H19-R* | TTCTAAGTGAATTACGGTGGGTG |
| *Parp3-F* | ATGGCTCCAAAACGAAAGGC |
| *Parp3-R* | TCCTCCTCTGTCCCTTGTCG |
| *GAPDH-F* | AGAAGGTGGTGAAGCAGGCATC |
| *GAPDH-R* | CGAAGGTGGAAGAGTGGGAGTTG |


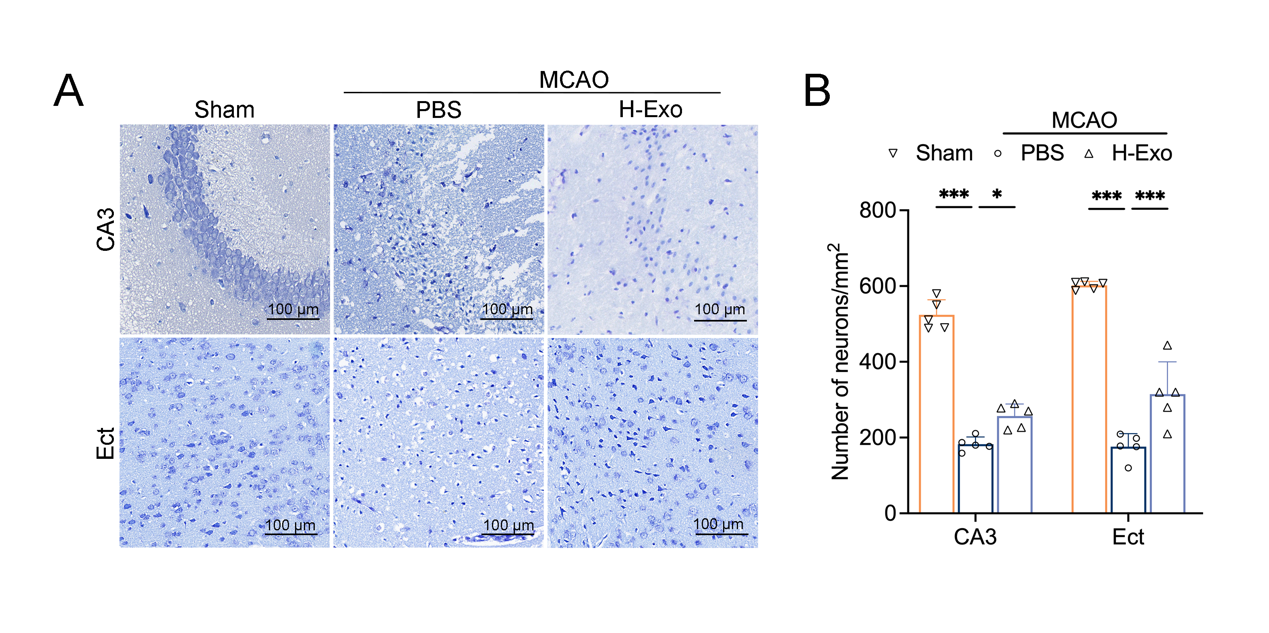


**Figure S1. H-Exo alleviates neuronal loss in the hippocampal CA3 and entorhinal cortex (Ect) regions after cerebral ischemia.**

(A) Representative Nissl staining of hippocampal CA3, and entorhinal cortex (Ect). (B) Quantification of surviving neurons per mm², revealing H-Exo–mediated neuroprotection across multiple brain regions. Scale bars = 100 μm. n = 5 mice per group. Two-way ANOVA. Data are presented as mean ± SD. **p* < 0.05; ***p* < 0.01; ****p* < 0.001.


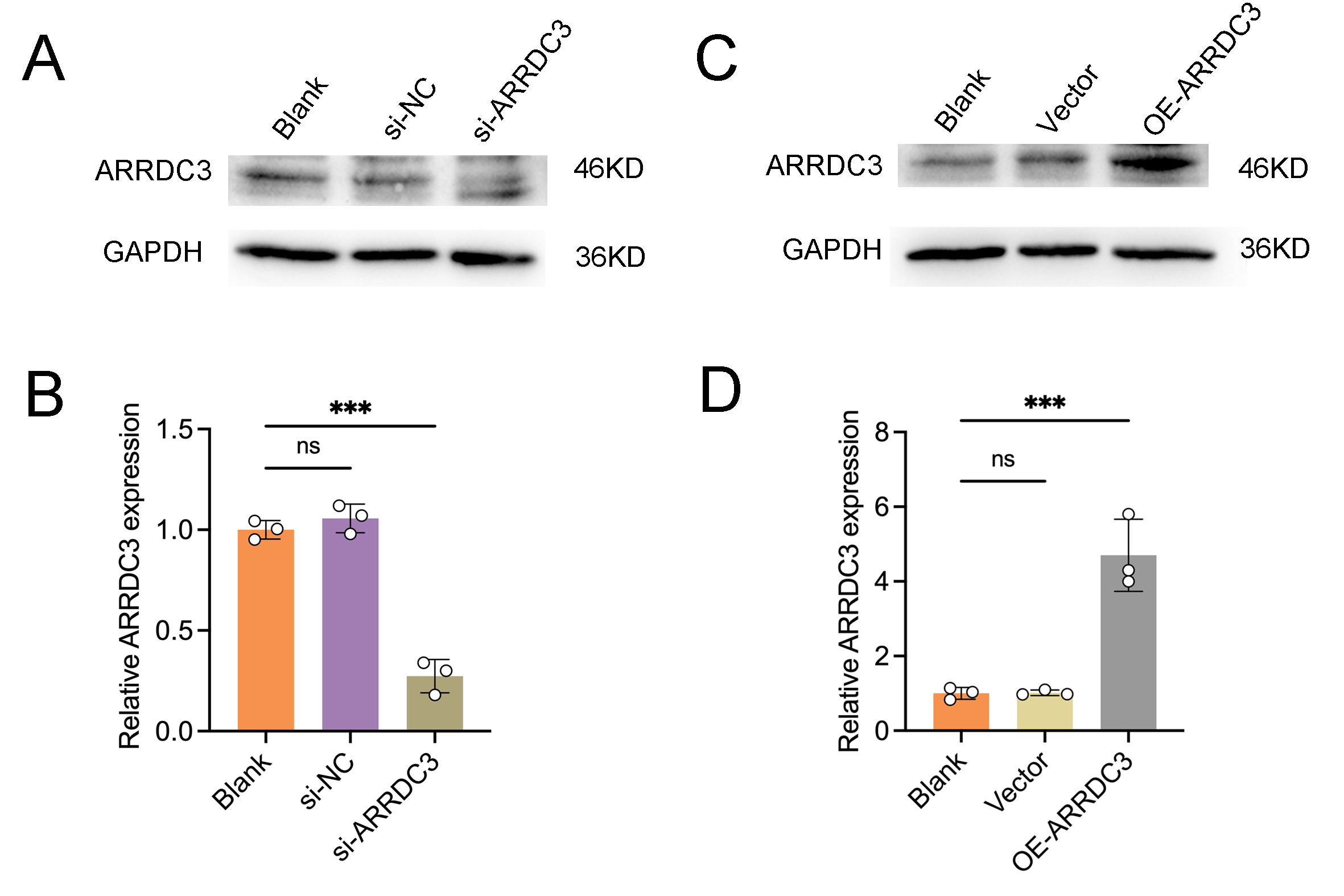


**Figure S2. Validation of ARRDC3 knockdown and overexpression.**

(A, B) Validation of ARRDC3 knockdown efficiency and quantification by Western blot. *n* = 3 independent experiments. (C, D) Validation of ARRDC3 overexpression efficiency and quantification by Western blot. *n* = 3 independent experiments. One-way ANOVA. Data are presented as mean ± SD. **p* < 0.05; ***p* < 0.01; ****p* < 0.001.


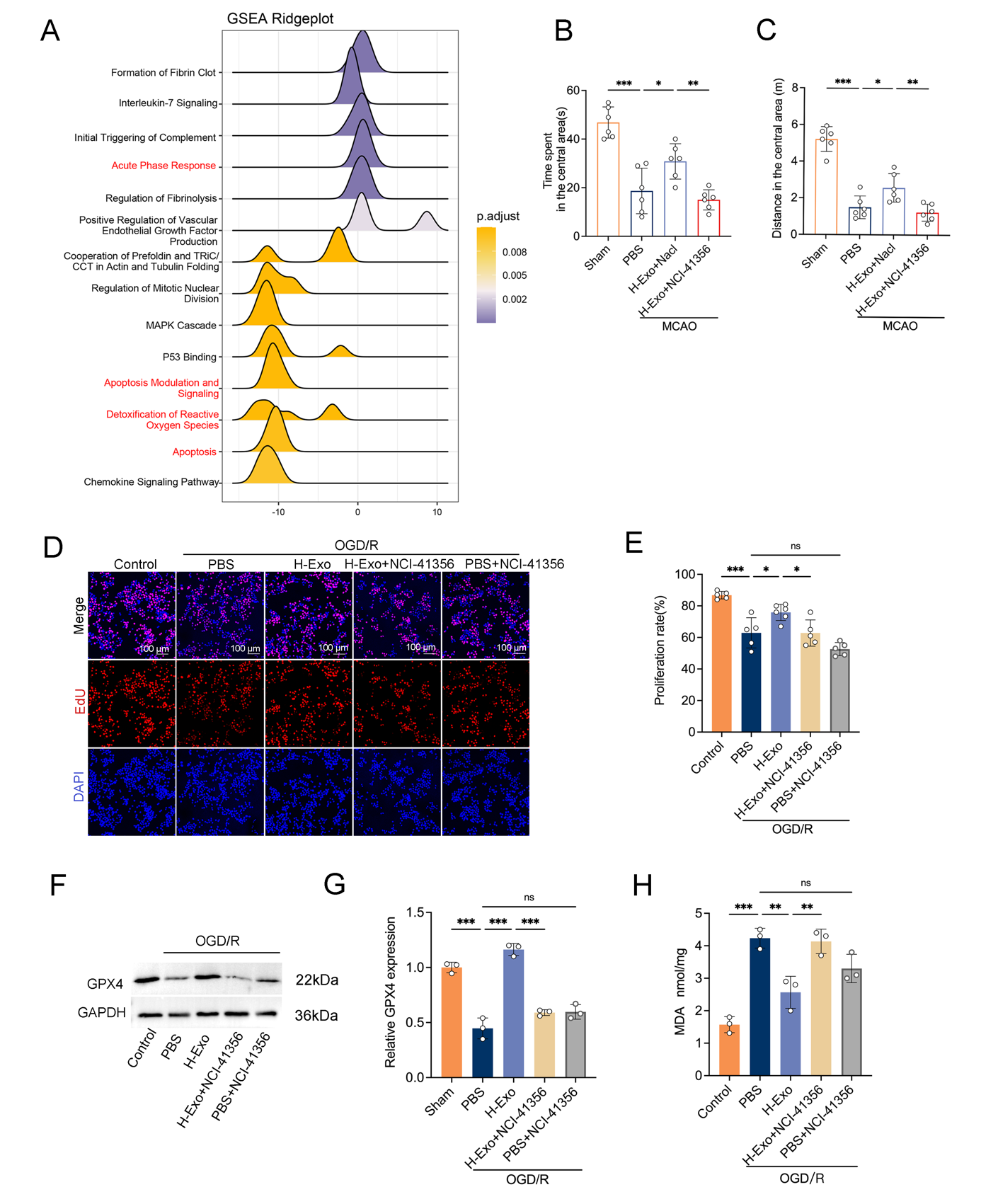


**Figure S3. NCI-41356 attenuates the H-Exo–mediated improvement of neuronal proliferation and ferroptosis suppression.**

(A) Gene set enrichment analysis (GSEA) ridgeplot showing representative biological pathways significantly regulated in H-Exo. (B, C) Open field test assessing locomotor and anxiety-like behaviors, including distance traveled in the central area (B), and time spent in the center area (C). (D, E) Representative EdU/DAPI staining images (D) and quantification of proliferating N2a cells (E). Scale bars = 100 μm. *n* = 5 biological replicates, data derived from *n* = 3 independent experiments. (F, G) Western blot analysis (F) and quantification of GPX4 protein expression (G) in each group. *n* = 3 independent experiments. (H) Quantification of malondialdehyde (MDA) levels in each group. *n* = 3 independent experiments. One-way ANOVA. Data are presented as mean ± SD. **p* < 0.05; ***p* < 0.01; ****p* < 0.001.
