# Supplementary material for: Targeting the ARRDC3–DRP1 Axis via hUMSC‐Derived Exosomal CRYAB for Neuroprotection in Cerebral Ischemia/Reperfusion Injury
Source: Adv Healthc Mater. 2026 Jan 19;15(14):e03803. doi: 10.1002/adhm.202503803 (PMC13068360; doi:10.1002/adhm.202503803)

Figure5H ARRDC3 and GAPDH

Note

a. The red dashed boxes indicate three independent replicates of ARRDC3 and GAPDH,

b. The blue dashed boxes show the corresponding low-exposure or non-exposed images used to visualize the molecular weight markers.

c. Short red lines on the left indicate the corresponding molecular weight markers shown on the right.

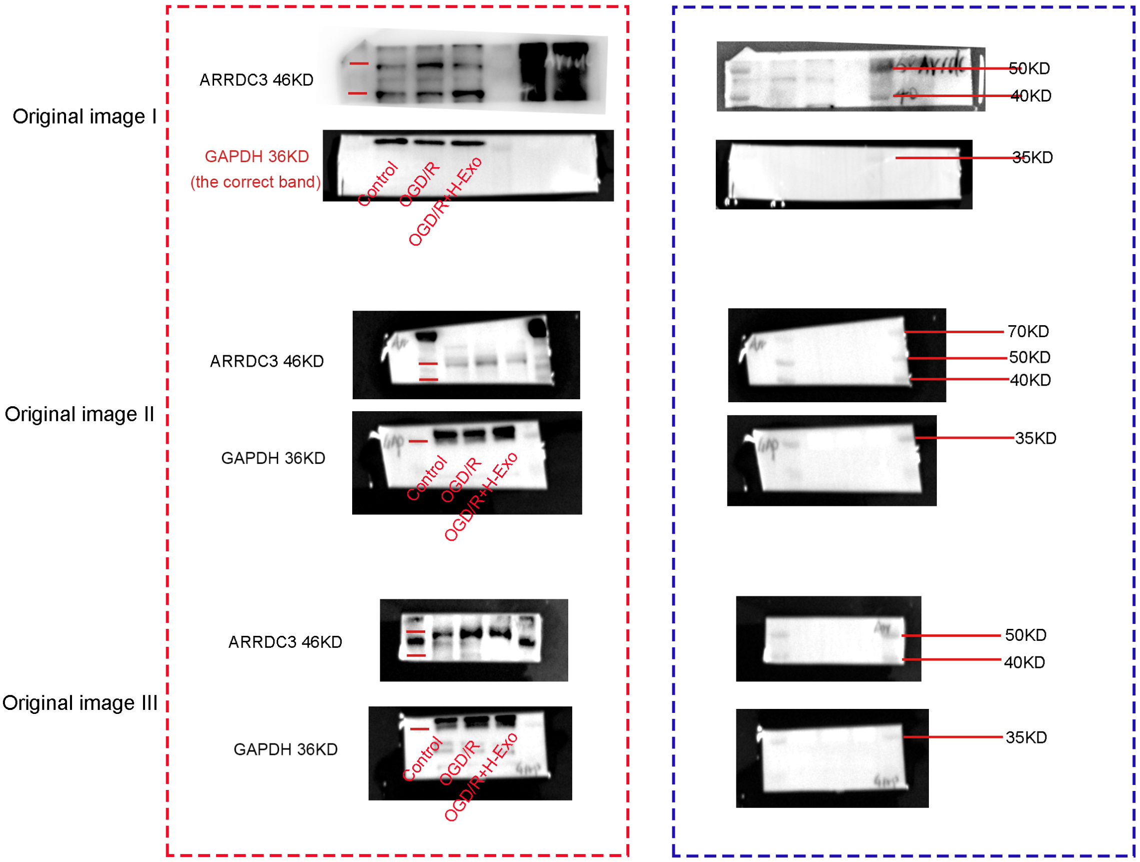

Supplementary Figure S2A

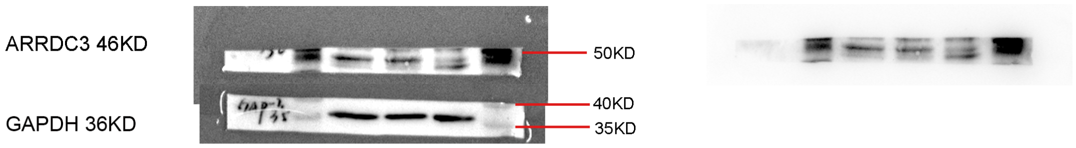

Supplement: Supplementary file 2 — Supporting File 2: adhm70791‐sup‐0002‐DataFile.pdf. [file ADHM-15-0-s002.pdf]
